# A Data-Driven Approach to Construct a Molecular Map of *Trypanosoma cruzi* to Identify Drugs and Vaccine Targets

**DOI:** 10.3390/vaccines11020267

**Published:** 2023-01-26

**Authors:** Swarsat Kaushik Nath, Preeti Pankajakshan, Trapti Sharma, Priya Kumari, Sweety Shinde, Nikita Garg, Kartavya Mathur, Nevidita Arambam, Divyank Harjani, Manpriya Raj, Garwit Kwatra, Sayantan Venkatesh, Alakto Choudhoury, Saima Bano, Prashansa Tayal, Mahek Sharan, Ruchika Arora, Ulrich Strych, Peter J. Hotez, Maria Elena Bottazzi, Kamal Rawal

**Affiliations:** 1 Centre for Computational Biology and Bioinformatics, Amity Institute of Biotechnology, Amity University, Noida 201303, Uttar Pradesh, India; 2 Texas Children’s Hospital Center for Vaccine Development, Departments of Pediatrics and Molecular Virology and Microbiology, Baylor College of Medicine, Houston, TX 77030, USA; 3 National School of Tropical Medicine, Baylor College of Medicine, Houston, TX 77030, USA; 4 Department of Biology, Baylor University, Waco, TX 76798, USA

**Keywords:** system biology, vaccine targets, pathways, gene ontology, chagas disease, *Trypanosoma cruzi*, drug target

## Abstract

Chagas disease (CD) is endemic in large parts of Central and South America, as well as in Texas and the southern regions of the United States. Successful parasites, such as the causative agent of CD, *Trypanosoma cruzi* have adapted to specific hosts during their phylogenesis. In this work, we have assembled an interactive network of the complex relations that occur between molecules within *T. cruzi*. An expert curation strategy was combined with a text-mining approach to screen 10,234 full-length research articles and over 200,000 abstracts relevant to *T. cruzi*. We obtained a scale-free network consisting of 1055 nodes and 874 edges, and composed of 838 proteins, 43 genes, 20 complexes, 9 RNAs, 36 simple molecules, 81 phenotypes, and 37 known pharmaceuticals. Further, we deployed an automated docking pipeline to conduct large-scale docking studies involving several thousand drugs and potential targets to identify network-based binding propensities. These experiments have revealed that the existing FDA-approved drugs benznidazole (Bz) and nifurtimox (Nf) show comparatively high binding energies to the *T. cruzi* network proteins (e.g., PIF1 helicase-like protein, trans-sialidase), when compared with control datasets consisting of proteins from other pathogens. We envisage this work to be of value to those interested in finding new vaccines for CD, as well as drugs against the *T. cruzi* parasite.

## 1. Background

Chagas disease is caused by the protist parasite *Trypanosoma cruzi*. It affects 6–7 million humans and a large number of animal species. The study of CD and *T. cruzi* is challenging, due to the complexity and unique characteristics of the parasite’s genome. For instance, 50% of the *T. cruzi* genome is composed of repeated sequences, such as transposable elements, microsatellites, and simple tandem repeats. It also includes surface molecules encoding genes, such as trans-sialidases, mucins, gp63s, and a large novel family (>1300 copies) of the mucin-associated surface protein (MASP) [1,2,3].

*T. cruzi* presents with a complex life cycle comprising four morphological stages: epimastigotes (EP), metacyclic trypomastigotes (MT), cell-derived trypomastigotes (CDT), and amastigotes (AM). During its life cycle, the parasite changes in morphology, metabolism, and gene expression, as it passes from the epimastigote replicative stage in the insect to the metacyclic trypomastigote form, which infects humans. *T. cruzi* appears to have a unique approach to evading both parasiticidal antibodies and T-cell recognition. It creates an antigenic variation by generating large families of genes encoding both surface and secreted proteins, many of which are expressed simultaneously. Previously, Nde et al., first elucidated the lamc1 sub-network interactome mobilised in the human coronary artery smooth muscle (HCASM) cells by *T. cruzi*. The authors presented the human extracellular matrix interactome network regulated by *T. cruzi* and its gp83 ligand that facilitates cellular infection [4]. These authors also reported that *T. cruzi* modulates the extracellular matrix (ECM) interactome to gain cell entry while evading the host immune system [4]. *T. cruzi* triggers gp83 receptors in the host cell via ERK1/2 to up-regulate LAMC1 which cross-talks to both *LGALS3* and *THBS1* to enhance cellular infection using selected parasite surface molecules, such as TcCRT and TC45 mucin [4]. Roberts et al. used proteomics and interaction data to generate constraint-based models, focusing on pathways of the central metabolism, such as glycolysis [5]. Researchers have also constructed genome-scale metabolic models of *T. cruzi* to investigate the variation in metabolic functions across the life cycle stages and to predict the stage-specific essential genes and reactions. These reactions are essential for the growth of *T. cruzi* and could potentially be drug targets, such as the cytosolic transketolase reaction, the mitochondrial alanine transport reaction, and the glycosomal glucokinase reaction [6].

Despite these efforts, there remains an urgent need to construct a molecular network of *T. cruzi* to understand the pathophysiology of trypanosome infection. Networks have played an important role in the understanding of complex diseases, such as obesity, atherogenesis, myocardial infarction, heart failure, Charcot–Marie–Tooth disease, and spinal muscular atrophy [7,8,9,10,11,12,13]. It has been proposed that pathogens that cause acute infections tend to target central hubs of the host’s cellular signaling networks, whereas pathogens causing chronic disease are more likely to target peripheral nodes [14]. Furthermore, the network pharmacology approach has been used to study “compound-protein/genes-disease” pathways, which can describe complexities among biological systems, drugs, and diseases. Thus, molecular networks provide significant avenues for the identification of drug targets [15,16,17], vaccine targets [18,19,20], and biomarkers [21,22,23].

Here, we present a molecular map of *T. cruzi* constructed from an extensive literature review using deep curation and text-mining techniques. We also conducted large-scale docking studies of benznidazole and nifurtimox against several thousand *T. cruzi* predicted protein structures using our new drug-dock approach to identify the important molecular targets of Bz and Nf in the *T. cruzi* molecular network. This work also identified other FDA-approved drug molecules, in the context of *T. cruzi*, and new strategies to identify drug targets using comparative approaches. 

## 2. Methods

A hybrid approach combining deep curation and text-mining strategies was used to curate the relevant information related to the *T. cruzi* pathways and molecules (genes, proteins, drugs, etc.) from published scientific articles. The abstracts were retrieved from literature databases, such as PubMed and Google Scholar using keywords, such as “*T. cruzi*” AND pathways name, “*T. cruzi* AND molecules (Gene, protein, and drug)”. Different networks were constructed using CellDesigner 4.4.2 [24], a modeling tool that enables us to graphically represent interactions using a well-defined and consistent graphical notation in the Systems Biology Markup Language (SBML) [25,26]. The schematic diagram for the complete methodology is shown in Supplementary Figure S1. The standard notation scheme for the graphical representation is provided in Supplementary Figure S2.

### 2.1. Extraction of the Literature Information Related to the T. cruzi Pathways

We extracted the names of the *T. cruzi* pathways using the keywords “*T. cruzi*” AND “pathway” on Google Scholar and PubMed. A total of 70,600 hits and 1142 hits were found on Google Scholar and PubMed, respectively. We shortlisted 46 unique pathways of *T. cruzi* from this total number of hits (Supplementary Table S1). All 46 pathways were classified, based on their function (Supplementary Table S2).

Further, we extracted the abstracts related to these 46 pathways from Google Scholar and PubMed using the keywords “*T. cruzi*” AND “<Name of pathway>” through deep curation strategies. Manual curation was performed using extracted abstracts and the text describing the gene/protein or any other molecules involved in the pathway was retrieved during the curation.

### 2.2. Retrieval of the T. cruzi Molecules and Their Function

**Figure 1:**
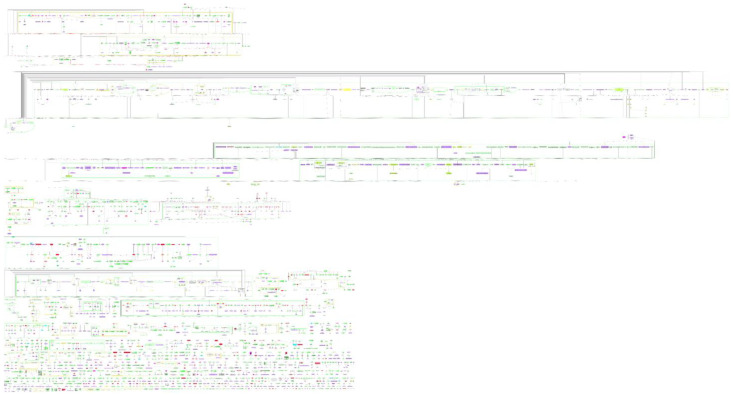
The network of the comprehensive molecular map which includes 1756 genes and 3109 proteins related to *T. cruzi*. The network is generated using CellDesigner v.4.4.2. Since the figure is very large in size, we are providing alternative links in the form of PNG on our website for the user to download and view (https://tinyurl.com/supplementary-data).

We retrieved 19,607 genes of *T. cruzi* CL Brener (*Tc*-CLB) from the NCBI database [27] (Supplementary Table S3). A total of 1756 unique genes were identified after removing the duplicate genes (Supplementary Table S4). To find out the function of the genes, an extensive literature search was carried out on PubMed and Google Scholar using the key terms “*T. cruzi*” AND “gene name”. All curated molecular evidence is summarized in Supplementary Table S5. In addition, 86,990 abstracts (Google Scholar-85,600, PubMed-1390, as of February 2021) were collected using the literature mining approach (Supplementary File S1). The 1756 unique genes with their known molecular interactions were used to construct a comprehensive molecular map (Figure 1).

Next, a total of 19,757 *Tc*-CLB proteins were retrieved from the UniProt database [28] (Supplementary Table S6). Out of this list, 3109 unique proteins were filtered manually (Supplementary Table S7). An extensive literature search was carried out in PubMed and Google Scholar using the key terms “*T. cruzi*” AND “protein name”. All curated molecular evidence with its interactions is outlined in Supplementary Table S8. A total of 103,868 abstracts (Google Scholar-96,300, PubMed-7568), as of February 2021, were screened to obtain the molecular information related to *T. cruzi* (Supplementary File S2). All unique proteins from the UniProt database, with their interaction information, were used for the construction of the molecular map.

### 2.3. Retrieval of the *T. cruzi* Drugs

For the construction of the *T. cruzi* drug network, we screened research articles manually, using the key terms “*T. cruzi*” AND “drug”. The 1693 abstracts with the terms “*T. cruzi*” AND “drug” were downloaded from Google Scholar and PubMed (Supplementary File S3). The molecular evidence describing the functions of the drugs were extracted and then used for the construction of the network using CellDesigner 4.4.2 [24]

#### 2.3.1. Networks Analysis

The network topological parameters were analyzed using a Cytoscape plugin called “Network Analyzer” [29]. It calculates several parameters, such as the number of nodes, number of edges, the average number of neighbors, network diameter, network radius, characteristic path lengths, clustering coefficient, network density, network heterogeneity, network centralization, and connected components. 

#### 2.3.2. Gene Ontology (GO) Analysis

The unique *T. cruzi* genes retrieved from various sources were used for the gene ontology analysis. The gene ontology was carried out for four categories i.e., biological process, molecular function, cellular component, and metabolic pathways. The enrichment analysis of the gene ontology (GO) [30], KEGG [31], and MetaCyC pathways [32] was performed for the *T. cruzi* genes using TriTrypDB v44 [33,34]. In TriTrypDB, the data is available for a wide range of kinetoplastids. For this analysis, we selected the *T. cruzi* organism to filter out only the *T. cruzi* genes (see https://tinyurl.com/TriTrypDB). Further, the significantly enriched GO terms were clustered, summarized, and visualized using REVIGO [35]. The GO terms and pathways enrichment were considered statistically significant when the false discovery rate or FDR was less or equal to 0.05.

#### 2.3.3. Homology Modelling of the *T. cruzi* Proteins

We retrieved the FASTA sequences of 19,607 *Tc*-CLB proteins from NCBI. Since only 127 crystal structures were available in the PDB for *T. cruzi*, we decided to construct three-dimensional (3-D) structures with the help of homology modelling. As a first step, we were able to find 4905 *Tc*-CLB proteins showing a similarity (≥35%) with the sequences available in the PDB. Next, we used the MODELLER tool [36], for the homology modeling of the proteins [37].

#### 2.3.4. Molecular Docking

We developed a new algorithm named drug-dock for the large-scale automated docking using Auto dock Vina [38]. This system is based upon Python, JavaScript, and Open Babel. The system also has a module for the prediction of protein structures using homology modeling [39] in case the target structure is not present in the PDB. We obtained the structural information on the genes/proteins implicated in *Tc*-CLB from the PDB. The structure information for Bz, Nf, orlistat, and aspirin, as well as other drugs was obtained from DrugBank [40] and their side effects were retrieved from the SIDER database. The PDB format of the protein structure was converted to PDBQT format before commencing the docking procedure. For the prediction of the binding site, we have incorporated the P2Rank tool [41] in the pipeline. The system performs the generation of a configuration file for each protein, converts the structure into PDBQT format, changes the ligand from SDF to PDBQT format using Open Babel [42], and performs molecular docking using Auto dock Vina. The system also converts the ligand’s 2d-SDF to 3d-SDF before converting it into PDBQT format. The obtained config files for the proteins and ligands in PDBQT format are provided as input to Auto Dock Vina. Auto Dock Vina generates a log file and an output file. The log file contains the binding energies of proteins and drugs, whereas the output file contains the 3D structures of the 10 highest-scoring poses (orientations) of each drug attached to the protein. During the process of parsing, the final result is generated in the form of a matrix. 

## 3. Results

### 3.1. General Features of the Comprehensive Molecular Map of T. cruzi

A total of 86,990 abstracts related to *Trypanosoma* genes (using keywords “*T. cruzi* AND genes”) were collected from Google Scholar (85,600 abstracts) and PubMed (1390 abstracts) (published till February 2021) (Supplementary File S1). Out of 19,607 genes available from NCBI (*Tc*-CLB dataset), only 1756 gene names were found to be unique in nature (Supplementary Tables S3 and S4). Next, we extracted the information about the function, as well as the interaction information for each gene (Supplementary Table S5).

Similarly, we retrieved 103,868 abstracts (Google Scholar and PubMed) using the keyword “*Trypanosoma cruzi* AND Protein” (February 2021) (Supplementary File S2). A list of the *Tc*-CLB proteins was collected from the UniProt database (19,757 proteins after removing the redundant hits [28]) (Supplementary Table S6). Further, we filtered the multi-copy proteins and hypothetical hits from the list to obtain 3109 unique protein names (Supplementary Table S7). Next, we extracted the interaction information for each of these proteins (Supplementary Table S8).

### 3.2. Features of the Comprehensive Map

The comprehensive molecular map comprises 2415 nodes and 1608 edges. The nodes include 190 genes, 1188 proteins, eight antisense RNAs, 201 complexes, 12 degraded, 48 drugs, 13 ions, 534 phenotypes, 22 RNAs, 42 unknown nodes, and 157 simple molecules (Supplementary Table S9). The edges were categorized into 1458 state transitions, five positive influences, 18 triggers, nine modulations, nine transport-related, seven translation-related, six transcription-related, one physical stimulation, and 92 others (Supplementary Table S10). The comprehensive molecular map is shown in Figure 1. It contains the molecules that are known to participate in important pathways, such as the ERK1/2 mitogen-activated kinase pathway, the glycogen synthase kinase 3 pathway, mTOR pathways, etc. We also found several housekeeping genes on the map, such as *bap1* (BRCA1 associated protein), *coq4* (coenzyme Q4), *dpy30* (Dpy-30 histone methyltransferase complex regulatory subunit), *mpc2* (mitochondrial pyruvate carrier 2), and *ndufv2* (NADH: ubiquinone oxidoreductase core subunit V2). The drugs reported as inhibitors of the *T. cruzi* growth were also included. These include Bz, tipifarnib (R115777), Nf, and leptomycin B.

### 3.3. Pathways of T. cruzi

**Figure 2:**
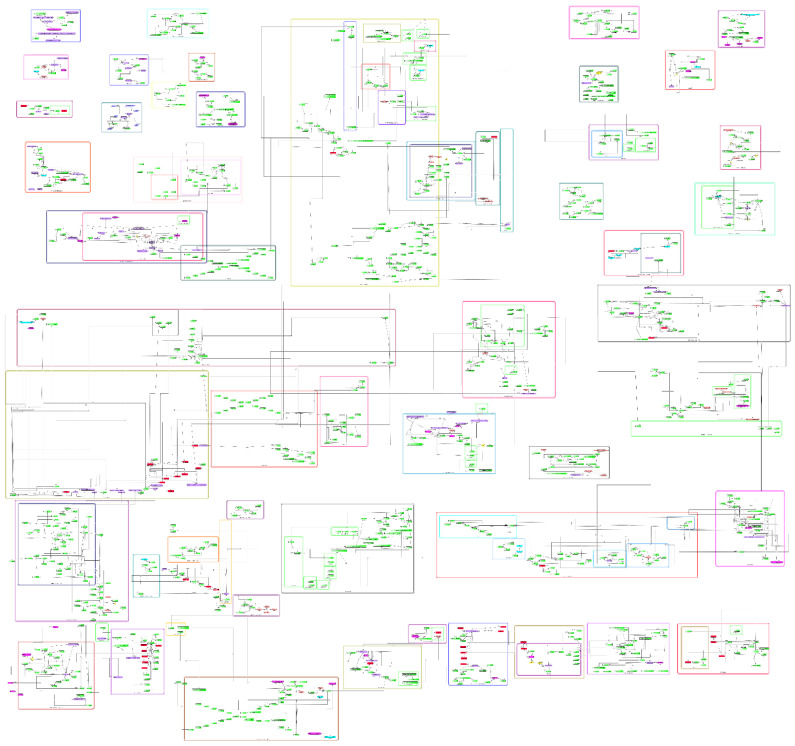
The network of molecules (gene, protein, drugs) involved in the 46 pathways. The network is generated in the CellDesigner software v.4.4.2. Since the figure is very large in size, we are providing alternative links in the form of PNG on our website for the user to download and view (https://tinyurl.com/supplementary-data).

**Figure 3:**
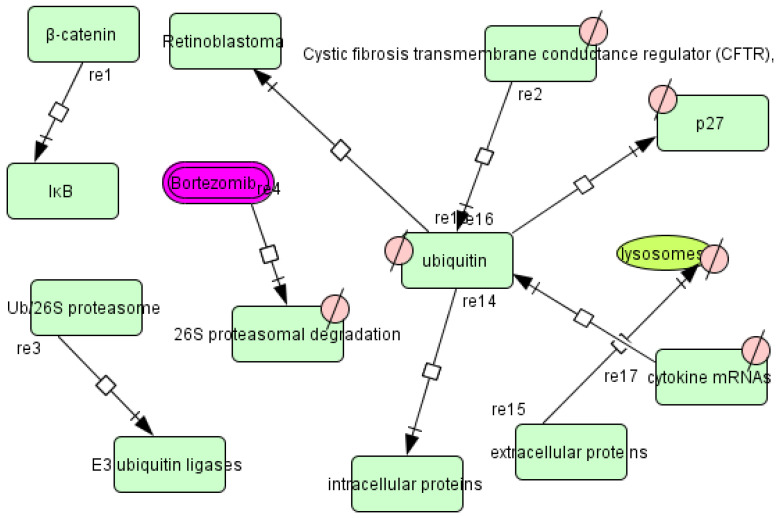
The network of the ubiquitin-proteasome pathway developed using the CellDesigner software (version—4.4.2).

Following the construction of the comprehensive map (see Section 3.2), we started collecting information on the pathways reported in the literature. Using text mining and deep curation, we found 46 unique pathway names in the literature (Supplementary Table S11). We extracted the molecules reported to be associated with these pathways. Further, we also extracted the molecular interaction information among the molecules involved in each pathway. A manually curated resource (database) containing papers or abstracts (with highlighted text to describe the role of the specific molecules) was assembled (Supplementary Table S1). A combined Systems Biology Markup Language (SBML) file was prepared for each pathway (Figure 2). In total, the pathways contained 1055 nodes that connect 43 genes, 838 proteins, one molecule involved in protein degradation, 37 drugs, 10 ions, 81 phenotypes, nine RNAs, and 36 simple molecules (Supplementary Table S12). These nodes are connected by 874 edges (Supplementary Table S13). We categorized these pathways into 17 major classes, including metabolic pathways, signaling pathways, degradative pathways, inflammatory pathways, etc. (Supplementary Table S2). As an example, we shall provide details of one of the constructed pathways (ubiquitin-proteasome pathway) in the next section (Figure 3).

Ubiquitination is an important process in eukaryotes and the ubiquitin-proteasome pathway enzymes are an important component of the protein degradation machinery. The process of degradation starts with the activation of ubiquitin (Ub), through the Ub-activating enzyme, E1, followed by the transfer of the activated Ub protein to Ub-conjugating enzymes (E2s) by the transacylation reaction. E2 transfers the Ub to the target protein substrates with the aid of the substrate-specific Ub ligases (E3s). The conjugation of a single Ub moiety is termed mono-ubiquitination and the subsequent conjugation of the Ub moieties leads to the formation of a polyubiquitin chain. This cascade of events leads the target substrate protein to the 26S proteasome for elimination [43]. To check the potential targets, we used molecular docking techniques to check the interactions of Bz and Nf (DB11820 and DB11989) against the molecules present in the ubiquitin-proteasome pathway. The molecules include glutathione peroxidase, ubiquitin/ribosomal protein S27a, ubiquitin-protein ligase, ubiquitin carboxyl-terminal hydrolase, 26S protease regulatory subunit, ubiquitin hydrolase, and ubiquitin-conjugating enzyme [44,45,46] (Supplementary Table S14).

### 3.4. *T. cruzi* Drugs and Network

**Figure 4:**
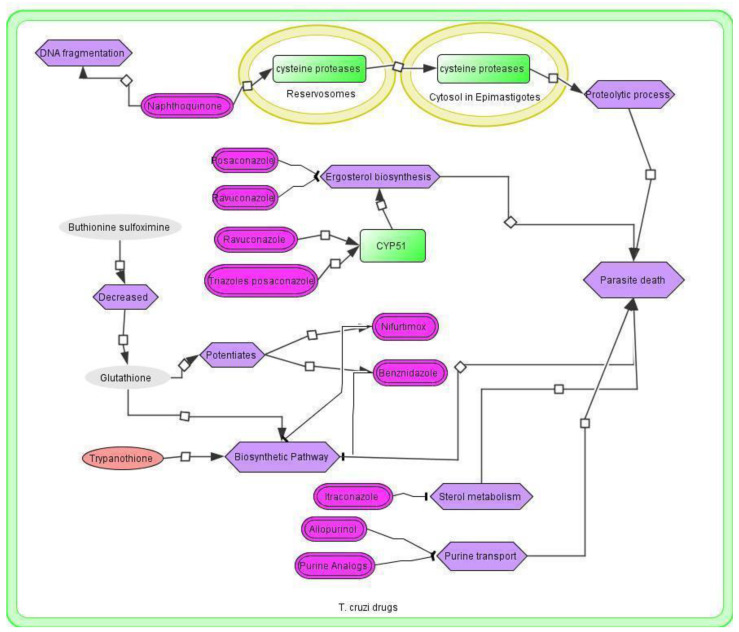
The drug network of *T. cruzi* developed using CellDesigner v.4.4.2.

An extensive literature search was carried out using PubMed and Google Scholar with the key terms “*T. cruzi* AND drug”. A total of 68,293 abstracts (Google Scholar 66,600 and PubMed 1693 hits published up to February 2021) were collected (Supplementary File S3). Each report was curated to obtain the molecular information related to the drug. The relevant line(s) or paragraph(s) was highlighted and used as evidence for building the drug network (Supplementary Table S15 and Figure 4). The drug network comprises 25 nodes including three proteins, 10 drugs, one simple molecule, five ions, nine phenotypes, and two unknown molecules that are connected via 26 edges (Supplementary Table S16). The edges represent interactions between each reactant or node. In this network, the interactions between reactants can be categorized into state transitions (19), and negative influences (Supplementary Table S17). In addition, we found 41 drugs for *T. cruzi* which are in different stages of development. For example, allopurinol, sulfasalazine, and thioridazine (TZD) are undergoing testing in the lab (experimental stages) [47,48,49,50] whereas posaconazole, ravuconazole, Nf, and Bz are in different phases of clinical testing [51,52,53,54] (Supplementary Table S18).

### 3.5. Application of the *T. cruzi* Drug Network

Treatment of CD is still limited to only two drugs, Bz and Nf [55,56]. Both are orally administered and can cause severe side effects, as well as long-term toxicity. Apart from these two drugs, naphthoquinone derivatives play an important role in DNA fragmentation, as well as in the release of cysteine proteases from reservosomes to the cytosol. This proteolytic process leads to parasite death [57] (Figure 2). Moreover, an antifungal—ravuconazole is a promising drug that is in clinical trials against CD, and used in combined therapy with Bz [58]. CYP51 (Sterol 14*α*-demethylase cytochrome P450) is an important enzyme with the trypanocidal activity responsible for ergosterol’s biosynthesis, that was identified in 1990. CYP51 inhibits the sterol synthesis, which is lethal to the parasite. Ravuconazole and posaconazole act through the coordination of nitrogen with heme iron into the binding cavity of CYP51 [59]. Studies on animal models found that posaconazole could be used for the treatment of acute and chronic CD [11]. The combination of Bz and itraconazole was shown to decrease the typical lesions (myocardial inflammation and fibrosis) associated with chronic CD and eliminate the parasites from the blood [12].

To check the interactions of various candidate drugs at the network level, we decided to use a drug dock algorithm. For instance, we docked Bz and Nf against the available 127 crystal structures of the *T. cruzi* proteins (Supplementary Table S19). We found that Nf is predicted to have the highest binding affinity with *T. cruzi* type B ribose 5-phosphate isomerase (TcRpiB) (−8.0 kcal/mol). During the literature curation, we found that the RpiB enzymes are present in the parasite whereas their homologs (RpiA) are absent. Further, TcRpiB turned out to be the only enzyme of the *T. cruzi* PPP (pentose phosphate pathway) which does not have a counterpart in higher eukaryotes. To check the potential impact of Bz and Nf on the PPP, we conducted a docking study against the members of the PPPs. As a comparison, we also studied the interactions of the controls (aspirin and orlistat) against the same targets. We found that the binding energy distributions are higher in the Bz and Nf study group, when compared with aspirin and orlistat. This holds, not only in the PPP of *T. cruzi*, but also in other pathways, suggesting differential binding preferences of Bz/Nf (Supplementary Table S20).

To study the potential side effects of Bz and Nf on humans, we conducted a large-scale docking study on the human proteome. For this, we collected 24,391 human proteins from the AlphaFold database [60] and performed docking with Bz. Due to technical issues, we could only dock 19,523 proteins with Bz. Interestingly, we identified biotin-protein ligase (Supplementary Table S21) as one of the top-ranking interactors of Bz (−9.1 kcal/mol of binding energy). Biotin is a water-soluble vitamin that belongs to the vitamin B complex and is an essential nutrient of all living organisms from bacteria to man [61]. In eukaryotic cells, biotin functions as a prosthetic group of enzymes, collectively known as biotin-dependent carboxylases that catalyze the key reactions in gluconeogenesis, fatty acid synthesis, and amino acid catabolism [61]. Biotin protein ligase (BPL) is required for the covalent attachment of biotin to biotin-dependent enzymes [62]. The clinical features of biotin deficiency include rashes, brittle hair, lethargy, hallucination, sleep disturbances, myalgia, and paraesthesia. The human biotin protein ligase (UniProt ID: P50747) is associated with glutamine deficiency and congenital phenotype [63] (OMIM database). Glutamine also contributes to the normal intestinal barrier function and can become deficient in some intestinal diseases, including Crohn’s disease, diarrheal illness, and short gut syndrome [64]. According to Viotti et al., the side effects of Bz vary from person to person [65]. The major side effects of Bz include insomnia, fatigue, anorexia, headache, furred tongue, gastrointestinal disturbances, skin rash, pruritus, erythema multiforme, and toxic epidermolysis [66,67]. We see a significant overlap between the clinical features of biotin deficiency and the side effects of Bz. There is a strong possibility that some of Bz’s side effects (i.e., gastrointestinal disturbance, sleep disturbances, etc.) can be linked to the off-target binding with BPL.

We further identified prostaglandin F synthase (PGF) (PDB: 4GIE) from *T. cruzi* bound to NADP, as another top-ranking target (−8.4 kcal/mol with benznidazole). The function of PGF is to catalyze the reduction of aldehydes and ketones to their corresponding alcohols. In humans, these reactions take place mostly in the lungs and the liver [68]. It is pertinent to note that PGF is involved in essential lipid-metabolism pathways in protists. Whereas in humans, PGF (Uniprot P42330) can interconvert active androgens, oestrogens, and progestins with their cognate inactive metabolites [69]. In humans, prostaglandins (PGs) E2, and PGF2α are produced in the endometrium and are important for menstruation and fertility [70]. Prostaglandin F2α synthase or old yellow enzyme (OYE), another NAD(P)H flavin oxidoreductase, similar to mitochondrial NADH-dependent type-I nitroreductase (NTR I), has been implicated in the activation pathway of other trypanocidal drugs, such as Nf but not Bz [71]. Different studies have shown that OYE was found to be downregulated in resistant parasites [72,73]. Murta et al. (2006) found that this protein was downregulated in resistant parasites, due to the deletion of three copies of the gene [73]. Likewise, by proteome analysis, it was found that OYE was under-expressed in resistant parasites [72]. Thus, PGF can be considered an important target for Bz. One of the reported side effects of Bz is hepatitis, which could be linked to the unwanted interaction of Bz with human PGF.

Next, we docked 1516 FDA-approved drugs with PGF, intending to find additional candidate drug molecules against CD (Supplementary Table S22). We found that the top three drug molecules were rifabutin, lurbinectedin, and amphotericin B. Further, we predicted 4905 structures of *T. cruzi* using homology modelling, since only 120 experimentally known structures were available in the PDB. Using this dataset, we found that the binding energy scores of Bz and Nf were significantly different from the binding energy scores of unrelated controls, aspirin, and orlistat (Welch’s T-test) (Supplementary Table S23). The top five ranking protein targets for Bz were found to be: hypothetical protein (XP_813710) (showing a similarity with the putative dynamin family), spermidine synthase (XP_816871), apurinic/apyrimidinic endonuclease (XP_816327), trans-sialidase (XP_802286), and peroxisome biogenesis factor 1 (XP_809676). Next, we used these top five targets to screen FDA-approved drugs to find potential drug candidates.

In a similar approach, the top five protein targets identified using Nf, were PIF1 helicase-like protein (XP_820585), hypothetical protein (XP_818590) (showing 99.77% similarity with RNA editing 3′ terminal uridylyl transferase 1 (*Trypanosoma cruzi*) (GenBank ID: PWV06812.1)), kinesin (XP_817032), ras-related protein Rab21 (XP_804862), and serine/threonine-protein kinase (XP_804576).

### 3.6. Therapeutic Implications of the *T. cruzi* Network

To check whether Bz/Nf produce their clinical effects, due to the preferential binding to several molecules listed in the *T. cruzi* network (N), rather than the proteomes of other pathogens, we used a dataset of 4905 *T. cruzi* protein structures (P). In addition, we created datasets of randomly selected protein structures from different pathogens (labelled as P1, P2,…Pn) as controls. We used proteins from *Plasmodium falciparum* (P1), *Mycobacterium tuberculosis* (P2), *Leishmania donovani* (P3), and *Salmonella typhi* (P4). We found that the binding energy distributions of Bz/Nf are significantly higher, when compared with the protein datasets selected from other pathogens (Welch’s T-test) (Supplementary Table S24). These results indicate that parasite-specific drugs (Bz/Nf) have specific binding affinities towards the parasite-specific proteins (i.e., *T. cruzi*). The reason could be attributed to the presence of specific types of residues and patterns/motifs in the binding sites of several parasite proteins.

Our research group has a long-standing interest in Tc24 (flagellar calcium-binding protein of 24 kDa). This protein has been proposed as a candidate for an immunotherapeutic vaccine, as well as a drug target [74]. Following the retrieval of the protein structure of Tc24 (accession ID: Q1L1I2_TRYCR) from AlphaFold [75], we conducted a large-scale docking study against Tc24 using drug molecules derived from the Zinc database [76] and a set of FDA-approved drug molecules. We found the drugs dutasteride, sirolimus, and candicidin to show the highest binding affinities against Tc24 (Supplementary Table S25).

Additionally, we conducted the molecular docking of Bz and Nf against Tc24 using Autodock Vina [38]. The PDB file for Tc24 was obtained from AlphaFold while the ligands (drugs) were obtained from DrugBank. The input PDBQT files for the protein and ligands were obtained from Autodock Vina [38]. The binding affinity for the top-scoring pose of Bz was observed as −6.9 kcal/mol while for Nf, it was observed as −6.7 kcal/mol. We also observed the distance of other modes from the best modes, in terms of the root mean square deviation (RMSD) (Supplementary Table S26 and Supplementary Figure S3).

### 3.7. Quantitative Analysis of the Networks

The topological analysis of all constructed networks was performed using a network analyzer tool [29] (Supplementary Table S27). The details were as follows:

(A) Clustering coefficient—If node A in a network is connected to node B, and B is connected to node C, A likely has a direct connection to C as well. The clustering coefficient can be used to quantify this phenomenon. It determines the average local neighborhood in a network [77], which varies frequently across the network [78]. If the clustering coefficient is near 0, the majority of nodes in the network have less than two neighbors, implying a tree-like topology [79]. The clustering coefficient value on our comprehensive map and modules is close to 0. The average clustering coefficient C(k), which indicates the metabolic network’s modularity [80], is another significant measure of the network’s structure. Using Network Analyzer [81], we discovered that our network’s average clustering coefficient was 0, indicating the presence of a tree-like structure;

(B) Network diameter is the maximum distance between two nodes. If the network is disconnected, the average of the maximum distances between the linked components is used to calculate the diameter. The diameters for the *T. cruzi* pathway network, molecular map network, and drug network were computed to be 28, 22, and 18 units, respectively;

(C) The characteristic path length is measured as an average number of edges dissociating any two nodes in the network. The pathway network has 1929 nodes with a path length of 11.73 units, the comprehensive map has 4016 nodes with a path length of 8.33 units, and the drug network has 51 nodes with a path length of 6.91 units;

(D) The average number of neighbors indicates the average connectivity of a node in the network. We observed 2.18 for our pathway network, 2.02 for the comprehensive molecular network, and 2.03 for the drug network;

(E) The network density determines compactness, which can be simply defined as the ratio of observed edges to the number of possible edges for the given network. The value ranges from 0 to 1, the closer the value, the denser and more cohesive the nodes in the network. We have computed the network density for our network and the average network density for all three networks is 0.027.

### 3.8. Gene Ontology Analysis of the *T. cruzi* Genes

To understand the biological functions of the *T. cruzi* genes used in the network construction, we performed a gene ontology analysis using TriTrypDB [33]. A total of 803 genes were recognized as belonging to the Trypanosomatidae class (Supplementary Table S28). We included only 72 genes that belong to two *T. cruzi* strains (CL Brener Esmeraldo-like (40), and Non-Esmeraldo-like (32)) (Supplementary Table S29). Based on function, the genes were enriched in a cellular component, molecular function, biological processes, and metabolic pathways. The cluster representation was performed using the REVIGO tool [35], which allows the clustering of semantically similar gene ontology terms, and labels each cluster with a single representative gene ontology term. The enriched metabolic pathway was considered statistically significant when the false discovery rate (FDR) was less than or equal to 0.05. The details of the enriched genes are provided in Supplementary Tables S30 and S31.

The *Tc*-CLB Esmeraldo-like genes were enriched in several molecular functions, such as cystathionine beta-synthase activity (GO:0004122), catalytic activity (GO:0003824), and metal ion binding (GO:0046872), different cellular components, such as the intracellular membrane-bounded organelle (GO:0043231), membrane-bounded organelle (GO:0043227), and mitochondrial inner membrane (GO:0005743), and the biological processes, such as alpha-amino acid biosynthesis (GO:1901607) or cellular amino acid biosynthesis (GO:0008652) (Supplementary Figures S4 and S5).

Similarly, the GO of *Tc*-CLB Non-Esmeraldo-like showed that the highest number of enriched genes were involved in different cellular components, such as the kinetochore (GO:0000776), chromosomal region (GO:0098687), and the site of double-strand breaks (GO:0035861), molecular functions, such as cystathionine beta-synthase activity (GO:0004122), catalytic activity (GO:0003824), and hydro-lyase activity (GO:0016836), and in biological processes, such as sulphur compound biosynthetic process (GO:0044272), cellular process (GO:0009987), cysteine metabolic process (GO:0006534), and cysteine biosynthetic process(GO:0019344), (Supplementary Figures S4 and S5). Many pathways, such as UDP-alpha-D-galactose biosynthesis, UDP-alpha-D-glucofuranose biosynthesis, D-galactose detoxification, and puromycin biosynthesis, were enriched in *Tc*-CLB Esmeraldo-like and Non-Esmeraldo-like (Supplementary Figure S6).

## 4. Discussion

This work combines the data from heterogeneous databases, including the literature, structure, and expression to construct a comprehensive map of *T. cruzi* molecules. It attempts to explain the interactions of the drug molecules in the context of networks. An effective drug molecule is expected to target the key molecules of the pathogen, as well as disrupt the key sections of its molecular network. Historically, molecular docking at a large scale has been deployed sparingly. Gao et al. used ~1100 targets [82]; Hui-Fang et al., used 1714 targets [10,83,84].

The major principle in drug discovery is to design maximally selective ligands to act on individual drug targets. However, several drugs act via the modulation of multiple proteins rather than single targets, suggesting the role of network-based therapeutics [85]. Here we performed the docking of trypanocidal drugs (Bz and Nf), control drugs (orlistat and aspirin), and 1500 FDA-approved drugs with *T. cruzi* network proteins (4905), as well as with the whole human proteome (19,523). We used our new drug repurposing pipeline to conduct large-scale docking [86]. Based on our prediction, we propose that the trypanocidal drugs show a preferential binding with *T. cruzi* proteins, as compared to the control drugs. It has been observed that benznidazole, not only binds to known targets, but also to several other targets of human proteins, which could explain some of the side effects of Bz. The overlap of the Bz side effects and biotin deficiency symptoms suggests a possibility of attenuating the side effects by the biotin administration [87,88]. Here, it is important to mention that another organic compound, “benzimidazole”, was studied by Woolley (1944). Benzimidazole derivatives have been reported to contain the trypanocidal activities [89]. Woolley (1944) reported that the similarity of the symptoms observed in animals receiving benzimidazole to those seen in biotin deficiency, suggested that the action of benzimidazole might be related to its structural similarity to biotin [90]. Both Bz and benzimidazole contain a common chemical nitrogen-containing ring.

We also observed that therapeutically unrelated drugs, i.e., orlistat and aspirin, displayed different binding patterns to the *T. cruzi* network proteins. Further studies are needed on other pathogens, which could explain the therapeutic effect of drugs at the network level. For example, similar studies could be conducted to study the binding of chloroquine against the proteome of malarial parasites. The other interesting potential application of our study is to compare the distribution of the binding energies of Bz/Nf in other related strains and species of *Trypanosoma*.

In our docking study, Nf was predicted to bind with *Tc*RpiB, with a binding affinity of −8.0 kcal/mol. *Tc*RpiB plays an important role in the pentose phosphate pathway (PPP). It is responsible for the production of nucleotide precursors and NADPH, which provide protection to trypanosomatids during oxidative stress [91]. A study performed by Loureiro et al. showed that RpiB silencing in *Trypanosoma brucei* reduced the in vitro growth of the parasites. Furthermore, RpiB silencing in the infected mice, exhibited lower parasitaemia and prolonged survival compared to control mice [92]. The absence of the RpiB enzyme in humans and its pivotal role in PPP makes it a potential chemotherapeutic target for trypanocidal drugs. RpiB is reported to be conserved among different *Trypanosoma* species [92]. Larkin et al. conducted a protein sequence alignment using ClustalW [93] and found a 67% identity for *T. brucei* RpiB versus *Tc*RpiB, and both proteins show no similarity with human ribose 5-phosphate isomerase A. Faria et al. compared the RpiB sequences of *L. infantum* (*Li*RPIB), *L. major* (*Lm*RPIB), *T. brucei* (*Tb*RPIB), and *T. cruzi* (*Tc*RPIB). They found that *Li*RPIB displays a 93% sequence identity with *Lm*RPIB and around 50% with RpiB from trypanosomes [94].

Biological systems are robust in the way that they restore the perturbations caused by drug treatments. One of the key avenues for a successful therapeutic drug or vaccine development is to overcome the biological robustness, maintained through positive or negative feedback loops of the drug/vaccine target proteins. The expression of several genes is believed to be altered during the disease phase. The genome-wide transcriptional profiling should enable us to specifically monitor the expression changes of the drug targets induced by their inhibitors or activators. The next version of this network will include the integrated genome-wide expression datasets, to study the perturbation induced by the therapeutic agent (drugs, vaccines, etc.) on *T. cruzi*.

Our research group has a long-standing interest in Tc24 (flagellar calcium-binding protein of 24 kDa). This protein has been proposed as a candidate for an immunotherapeutic vaccine, as well as a drug target. We also conducted a large-scale docking study against Tc24 using drug molecules derived from the Zinc database and a set of FDA-approved drug molecules. We found the drugs dutasteride, sirolimus, and candicidin have shown the best binding affinities against Tc24. (Supplementary Table S25).

Considering the wide variety of factors affecting the CD pathophysiology, we believe that a *T. cruzi* comprehensive map will act as a useful tool to provide information extracted from gene expression experiments, protein-protein interaction data, drug information, and clinical data information. Moreover, this will also advance our research group’s effort to use the ‘systems vaccinology’ approach to develop safe and effective vaccines against neglected tropical diseases, including CD [95]. For instance, Querec et al., 2008 used a systems biology approach to identify the early gene ‘signatures’ that predicted the immune responses in humans vaccinated with the yellow fever vaccine YF-17D [96]. Similarly, Nakaya et al. (2011) found that in subjects vaccinated with the trivalent inactivated influenza vaccine, early molecular signatures correlated with and could be used to accurately predict later antibody titers in two independent trials [97]. Li et al., (2014) performed a large-scale network integration of publicly available human blood transcriptomes and systems-scale databases in specific biological contexts, and deduced a set of transcription modules in the blood [98]. Those modules revealed distinct transcriptional signatures of antibody responses to different classes of vaccines, which provided key insights into primary viral, protein recall, and anti-polysaccharide responses [98]. These examples demonstrate the power of network-based approaches to predict immunogenicity and provide new mechanistic insights about vaccines.

## 5. Conclusions

A formalized depiction of the biological pathways is increasingly recognized as a crucial requirement for the exchange of the pathway data, modeling of their activity, and systems-level interpretation of biological data. However, there are just a handful of examples of large pathway diagrams constructed using a formalized graphical modeling language, such as SBML. The model of the *T. cruzi* pathways presented here is the most comprehensive pathway of its kind published to date. Although a time-consuming and laborious exercise, the act of converting the literature-derived knowledge into a formalized computational model is essential if we wish to truly gain a systems-level understanding of any cellular system. The *T. cruzi* pathways presented here summarize the results of years of investigations and have allowed the thorough testing of the notation system used to depict it. Furthermore, we performed large-scale molecular docking using our in-house pipeline to identify potential vaccine and drug targets. Our analysis could identify potential targets, such as type B ribose 5-phosphate isomerase, flagellar calcium-binding protein, and prostaglandin F synthase. These proteins are extensively reported in the literature as potential vaccine and drug targets [91,99,100,101]. 

## Data Availability

Data is available in the supplementary materials. In addition, data can be accessed on a dedicated website (https://tinyurl.com/Tcruzipathwaymapx).

## Supplementary Materials

The following supporting information can be downloaded at: https://www.mdpi.com/article/10.3390/vaccines11020267/s1, Supplementary Figure S1: The schematic diagram describing the flowchart to achieve the methodology, Supplementary Figure S2: The standard notations for the graphical representation of species and edges in cell designer v.4.4.2, Supplementary Figure S3: Docking of Tc24 protein with Benznidazole and Nifurtimox using AutoDock Vina. (a. Benznidazole, b. Nifurtimox). The top scoring pose of docking affinity against Tc24 for Benznidazole is −6.9 kcal/mol while the docking affinity for Nifurtimox is −6.7 kcal/mol, Supplementary Figure S4A: *Trypanosoma cruzi* CL Brener Esmeraldo-like. Enriched GO terms of genes (a. Biological process, b. Cellular component, c. Molecular function). The size of the dots and their color gradient (according to the legend) reflects the log10 value of the *p* value of each GO term, larger dots represent higher significance (all *p* values < 0.05). X and Y axes represent semantic spaces which have no intrinsic meaning. REVIGO uses multi-dimensional scaling to reduce the dimensionality of a matrix of the GO terms’ pairwise semantic similarities. This results in semantically similar GO terms remaining close together in the plot, Supplementary Figure S4B: *Trypanosoma cruzi* CL Brener Non-Esmeraldo-like. Enriched GO terms of genes (a. Biological process, b. Cellular component, c. Molecular function). The size of the dots and their color gradient (according to the legend) reflects the log10 value of the *p* value of each GO term, larger dots represent higher significance (all *p* values < 0.05). X and Y axes represent semantic spaces which have no intrinsic meaning. REVIGO uses multi-dimensional scaling to reduce the dimensionality of a matrix of the GO terms’ pairwise semantic similarities. This results in semantically similar GO terms remaining close together in the plot, Supplementary Figure S5A: *Trypanosoma cruzi* CL Brener Non-Esmeraldo-like. “Interactive graph” view of REVIGO (a. Biological process, b. Cellular component, c. Molecular function). Bubble color indicates the user-provided *p*-value. Highly similar GO terms are linked by edges in the graph, where the line width indicates the degree of similarity, Supplementary Figure S5B: *Trypanosoma cruzi* CL Brener Esmeraldo-like. “Interactive graph” view of REVIGO (a. Biological process, b. Cellular component, c. Molecular function). Bubble color indicates the user-provided *p*-value. Highly similar GO terms are linked by edges in the graph, where the line width indicates the degree of similarity, Supplementary Figure S6: Gene ontology (GO) classification, KEGG/MetaCyc analysis of the *T. cruzi* genes (a.) *T. cruzi* CL Brener Esmeraldo like (b.)*T. cruzi* CL Brener Non-Esmeraldo like). The percentage regulated genes based on GO annotations and KEGG/MetaCyc pathways. (c) word cloud graph depicting enriched Metabolic Pathway (1) *Trypanosoma cruzi* CL Brener Esmeraldo-like (2) *Trypanosoma cruzi* CL Brener Non-Esmeraldo-like, Supplementary Table S1a: List of all the curated evidence for the construction of *T. cruzi* map from 46 pathways. This table provides information about 46 pathways derived from literature; constituent molecules and the literature evidence. The information is presented in 4 columns: Name; Molecule; Evidence and PMID/LINK. The first column ‘Name’ provides information about the name of pathways reported in the literature; The second column ‘Molecule’ lists the specific molecule involved in the given pathway. The third column ‘Evidence’ shows specific lines/statements/paragraphs from the article/research paper that provides evidence for the role of the molecule in the specific pathway. The last column ‘PMID/LINK’ provides PMID or hyperlink of the paper from where evidence has been collected, Supplementary Table S1b: List of curated evidence of different biological molecule interactions for the construction of 46 *T. cruzi* pathway map. This table shows a curated list of biological molecule interactions evident to construct the 46 *T. cruzi* pathway map. [Molecule 1 = first molecule of the interaction; Molecule 2 = second molecule of the interaction; Evidence = Statement from the article that provides evidence for the role of the molecule in the specific pathway; Interactions = type of interaction between molecule 1 and molecule 2; PMID/LINK = PMID or link of the paper from where evidence has been collected], Supplementary Table S2a: List of curated pathways and their classifications. This table shows the classification of curated pathways and the sources from where they were extracted. This table has information in 4 columns: Name; Classification; Source 1, and Source 2. The first column ‘Name’ provides information about the name of pathways reported in the literature about *T. cruzi*; The second column ‘Classification’ lists the classes of pathways. The third column ‘Source 1’ is the first source of information from where pathways are extracted. The last column ‘Source 2’ is the second source of information from where pathway classifications are extracted, Supplementary Table S2b: List of curated pathways and their classifications. This table holds the list of curated pathways and their classifications based on the organism and host interaction. [Pathway name = Name of the pathway; Pathway from Organism = Source organism of the pathway; Type of pathway = The type of a specific pathway], Supplementary Table S3: The list of *Tc*-CLB genes retrieved from the NCBI database. This table has information in 8 columns: Tax_id, Organism name, GeneID, CurrentID, Status, Symbol, Aliases, and Description. The first column ‘Tax_id’ is the taxonomic ID of *T. cruzi*; The second column ‘Organism name’ is the name of the organism with strain detail. The third column ‘GeneID’ is the specific ID provided to the gene by NCBI. The fourth column ‘CurrentID’ is the ID provided by NCBI. ‘Status’ gives the current status of the gene in NCBI. ‘Symbol’ is the gene symbol. ‘Aliases’ gives the available aliases of gene symbols. The last column ‘Description’ provides the name of the gene, Supplementary Table S4: The list of unique *Tc*-CLB genes used for the construction of the comprehensive molecular map, Supplementary Table S5a: The List of curated molecular evidence of *T. cruzi* genes with their interactions. This table has information in 5 columns: S.No., Molecule Layer 1 (*T. cruzi*), Molecule layer 2 (Human/Mice), Evidence, and PMID/LINK. The first column ‘S.No.’ is the serial number; The second column ‘Molecule layer 1 (*T. cruzi*)’ is the first molecule from *T. cruzi* in the interaction. The third column ‘Molecule layer 2 (Human/Mice)’ is the second molecule from Humans or Mice in the interaction. The fourth column is ‘Evidence’ which shows specific lines/statements/paragraphs from the article/research paper that provides evidence for the role of the molecule in the specific pathway. The last column ‘PMID/LINK’ provides a PMID or hyperlink of the paper from where evidence has been collected, Supplementary Table S5b: The Summary of the source of the information. [Name = Name of the organism whose information is collected; Keyword = phrases used for literature search; PubMed hits = Number of hits found in Pubmed; Google scholar hits; Number of hits found in Google Scholar; Date = till what date data is collected], Supplementary Table S6: The list of *Tc*-CLB proteins retrieved from the Uniprot database. This table has information in 4 columns: Entry, Protein names, Gene names, and Organism. The first column ‘Entry’ provides UniProt ID for a specific protein entry; The second column ‘Protein names’ lists the names of the proteins. The third column ‘Gene names’ provides the names of the genes that encode the protein. The last column ‘Organism’ provides the name of the organism from which the protein was extracted, Supplementary Table S7: The list of unique *T. cruzi* proteins used for the construction of the comprehensive molecular map [n = 3109], Supplementary Table S8a: The list of curated molecular evidence of *T. cruzi* proteins with their interactions. This table has information in 4 columns: Protein, Molecule 2 (Human/Mice), Evidence, and PMID/Link. The first column ‘Protein’ provides the name of the protein from *T. cruzi*; The second column ‘Molecule2 (Human/Mice)’ lists the second molecule from Humans or Mice in the interaction. The third column ‘Evidence’ shows specific lines/statements/paragraphs from the article/research paper that provides evidence for the role of the molecule in the specific pathway. The last column ‘PMID/LINK’ provides a PMID or hyperlink of the paper from where evidence has been collected, Supplementary Table S8b: The Summary of the source of the information. [Name = Name of the organism whose information is collected; Keyword = phrases used for literature search; PubMed hits = Number of hits found in Pubmed; Google scholar hits; Number of hits found in Google Scholar; Date = till what date data is collected], Supplementary Table S9: The list of nodes involved in the comprehensive molecular map of *T. cruzi*. This table has information in 12 columns: class, id, name, compartment, positionToCompartment, included, quantity type, initialQuantity, substanceUnits, hasOnlySubstanceUnits, b.c., and constants. The first column ‘class’ provides molecule type; The second column ‘id’ is the specific ID assigned to each entry. The third column ‘name’ lists the name of the molecule. ‘compartment’ is the compartment where the protein belongs; ‘positionToCompartment’ provides information on where the molecule is located. ‘included’ provides information on interactions. ‘quantity’ provides selected option for quantity an entry in terms of either amount as molecular/item count or concentration as units of substance/units of size; ‘initialQuanity’ list the value set as initial quantity to run the simulation; ‘substanceUnit’ provides the unit assigned to each entry. ‘hasOnlySubstance’ gives a Boolean (True or False) on if species quantity always be as substance or substance/size; ‘b.c.’ is the boundary condition, which is a boolean (True or False) on should a rate of change equation be constructed for the species based on the system of reactions. The last column ‘constants’ is a boolean (True or False) on if the species quantity is constant, Supplementary Table S10: List of edges involved in the comprehensive molecular map of *T. cruzi*. This table has 7 columns: type; id; reversible; fast; reactants; products and modifiers. The first column ‘type’ provides information on the different types/categories of the reactions involved in the molecular map. The second column ‘id’ is the specific ID assigned to each entry. The third column ‘reversible’ gives a Boolean (True or False) on if the reaction is reversible or not. The fourth column ‘fast’ provides a Boolean (True or False) on if the reaction is fast or not. The fifth column ‘reactants’ lists unique molecule IDs (from Supplementary Table S9) of the reactant molecules in the reaction. The sixth column ‘products’ lists unique molecule IDs (from Supplementary Table S9) of the product formed in the reaction. The last column ‘modifiers’ lists unique molecule IDs (from Supplementary Table S9) of molecules that act as modifiers in the reaction, if any, Supplementary Table S11: The list of the total number of curated abstracts retrieved from Google scholar and PubMed against 46 unique *T. cruzi* pathways. This table has 5 columns: Name; Keyword Used; Google scholar hits; PubMed hits and Date. The first column ‘Name’ provides the list of *T. cruzi* pathways collected from literature and used for the construction of the molecular map. The second column ‘Keyword Used’ provides information about the key terms that were used for the literature search. The third column ‘Google scholar hits’ contains the number of abstracts/hits obtained from Google Scholar using the respective keyword. The fourth column ‘PubMed hits’ contains the number of abstracts/hits obtained from PubMed using the respective keyword. The last column ‘Date’ list the date till when the literature was extracted, Supplementary Table S12: The list of nodes involved in the network of *T. cruzi* pathway. This table has information in 10 columns: class, id, name, compartment, positionToCompartment, quantity type, initialQuantity, hasOnlySubstanceUnits, b.c., and constants. The first column ‘class’ provides molecule type; The second column ‘id’ is the specific ID assigned to each entry. The third column ‘name’ lists the name of the molecule. ‘compartment’ is the compartment where the molecule belongs; ‘positionToCompartment’ provides information on where the molecule is located. ‘quantity type’ provides a selected option for the quantity of an entry in terms of either amount as molecular/item count or concentration as units of substance/units of size; ‘initialQuanity’ list the value set as initial quantity to run the simulation; ‘hasOnlySubstance’ gives a Boolean (True or False) on if species quantity always be as substance or substance/size; ‘b.c.’ is the boundary condition, which is a boolean (True or False) on should a rate of change equation be constructed for the species based on the system of reactions. The last column ‘constants’ is a boolean (True or False) on if the species quantity is constant, Supplementary Table S13: List of edges involved in the network of *T. cruzi* pathways. This table has 7 columns: type; id; reversible; fast; reactants; products and modifiers. The first column ‘type’ provides information on the different types/categories of the reactions involved in the molecular map. The second column ‘id’ is the specific ID assigned to each entry. The third column ‘reversible’ gives a Boolean (True or False) on if the reaction is reversible or not. The fourth column ‘fast’ provides a Boolean (True or False) on if the reaction is fast or not. The fifth column ‘reactants’ lists unique molecule IDs (from Supplementary Table S9) of the reactant molecules in the reaction. The sixth column ‘products’ lists unique molecule IDs (from Supplementary Table S9) of the product formed in the reaction. The seventh column ‘modifiers’ lists unique molecule IDs (from Supplementary Table S9) of molecules that act as modifiers in the reaction, if any, Supplementary Table S14: The docking of proteins from the Ubiquitin Proteasome pathway with Nifurtimox and Benznidazole. This table has 3 columns: Protein name, DB11820 (Nifurtimox) and DB11989 (Benznidazole). The first column ‘Protein name’ contains the list of proteins present in the ubiquitin-proteasome pathway which were docked against Nifurtimox and Benznidazole. The second column ‘DB11820 (Nifurtimox)’ provides the binding affinities of the various pathway proteins when docked against Nifurtimox. The third column ‘DB11989 (Benznidazole)’ provides the binding affinities of the various proteins when docked against Benznidazole, Supplementary Table S15a: List of curated molecular evidence of *T. cruzi* drugs with its interactions. This table has 6 columns: Name; Keyword; PubMed hits; Google scholar hits; Date; and Total. The first column ‘Name’ provides information on the name of the molecule. The second column ‘Keyword’ provides information about the key terms that were used for the literature search. The third column ‘PubMed hits’ contains the number of abstracts obtained by using PubMed. The fourth column ‘Google Scholar hits’ contains the number of abstracts obtained by Google Scholar. The fifth column ‘Date’ provides information about the date on which the literature search was performed/completed. The sixth column ‘Total’ provides the sum total of hits from PubMed and Google Scholar combined, Supplementary Table S15b: List of curated molecular evidence of *T. cruzi* drugs with its interaction. This table has 4 columns: DRUG; EVIDENCE; LINK/PMID and DATE. The first column ‘DRUG’ provides the name of the drug. The second column ‘EVIDENCE’ shows specific lines/statements/paragraphs from the article/research paper that provides evidence of the interaction between the drug and *T. cruzi* molecules. The third column ‘LINK/PMID’ provides PMID of the paper from where the evidence has been collected. The fourth column ‘Date’ provides information about the date on which the literature search was performed/completed, Supplementary Table S15c: List of curated molecular evidence of *T. cruzi* drugs with its interaction. This table has 5 columns: DRUG, MOLECULE, EVIDENCE; LINK and DATE. The first column ‘DRUG’ provides a list of the drug names. The second column ‘MOLECULE’ provides the name of the molecule that interacts with the drug; The third column ‘EVIDENCE’ shows specific lines/statements/paragraphs from the article/research paper that provides evidence of the interaction between drugs and molecules. The fourth column ‘LINK’ provides a hyperlink of the paper from where the evidence has been collected. The fifth column ‘Date’ provides information about the date on which the literature search was performed/completed, Supplementary Table S16: List of nodes involved in the comprehensive molecular map of drugs. This table has 10 columns: class; id; name; compartment; positionToCompartment; quantity type; initialQuantity; hasOnlySubstanceUnits; b.c. and constants. The first column ‘class’ provides information on the various categories of nodes present in the network i.e., drug, protein, etc. The second column ‘id’ is the specific ID assigned to each entry. The third column ‘name’ contains the names of the different molecules. The fourth column ‘compartment’ is the compartment where the molecule belongs. The fifth column ‘positionToCompartment’ provides information on where the molecule is located. The sixth column ‘quantity type’ provides a selected option for the quantity of an entry in terms of either amount as molecular/item count or concentration as units of substance/units of size. The seventh column ‘initialQuantity’ contains the value set as initial quantity to run the simulation. The eighth column ‘hasOnlySubstanceUnits’ gives a Boolean (True or False) on if species quantity always be as substance or substance/size. The ninth column ‘b.c.’ is the boundary condition, which is a boolean (True or False) on should a rate of change equation be constructed for the species based on the system of reactions. The last column ‘constants’ is a boolean (True or False) on if the species quantity is constant, Supplementary Table S17: List of edges involved in the comprehensive molecular map of drugs. This table has 6 columns: type; id; reversible; fast; reactants and products. The first column ‘type’ provides information on the type of reactions between reactants i.e., state transitions or influences. The second column ‘id’ is the specific ID assigned to each entry. The third column ‘reversible’ helps us to know whether the reaction involved is reversible or not. The fourth column ‘fast’ provides information about the speed of the reaction, whether it’s fast or slow. The fifth column ‘reactants’ contains the IDs given to the reactant(s) involved. The last column ‘products’ contains the IDs given to the product(s) involved, Supplementary Table S18: The list of drugs used in the network with its experimental status. This table has 5 columns: Drug Name; PMID/link; Experimental status of the drug; Drug Bank ID and FDA-approved. The first column ‘Drug Name’ provides the list of various *T. cruzi* drugs used in the network and which are in different stages of development; The second column ‘PMID/link’ contains the link for the paper from which the data was collected; The third column ‘Experimental status of the drug’ provides the experimental status of the drug; The fourth column ‘DrugBank ID’ contains the IDs of the drugs that can be used to access the details of the drugs from the DrugBank Database; The last column ‘FDA Approved’ provides information on the developmental stage of the drugs i.e., approved, investigational, etc., Supplementary Table S19a: The binding affinity of the crystal structure of *T. cruzi* docked with Nifurtimox (DB11820). This table has 3 columns: PDB ID; Binding affinity and Protein name. The first column ‘PDB ID’ provides the PDB IDs corresponding to the available crystal structures of the 127 proteins of *T. cruzi*; The second column ‘Binding affinity (kcal/mol)’ provides the binding affinities of the 127 *T. cruzi* proteins with Nifurtimox; The third column ‘Protein name’ contains the names of the proteins corresponding to the PDB IDs, Supplementary Table S19b: The binding affinity of the crystal structure of *T. cruzi* docked with Benznidazole (DB11989). This table has 3 columns: PDB ID; Binding affinity and Protein name. The first column ‘PDB ID’ provides the PDB IDs corresponding to the available crystal structures of the 127 proteins of *T. cruzi*; The second column ‘Binding affinity (kcal/mol)’ provides the binding affinities of the 127 *T. cruzi* proteins with Benznidazole; The third column ‘Protein name’ contains the names of the proteins corresponding to the PDB IDs, Supplementary Table S19c1: Comparison of binding affinities (kcal/mol) obtained from docking against Nifurtimox (DB11820) with top 10 crystal structure and solved structure. This table has 3 columns: PDB ID; Binding score and Protein name. The first column ‘PDB ID’ provides the PDB IDs corresponding to the top 10 crystal structures of the *T. cruzi* proteins. The second column ‘Binding affinity (kcal/mol)’ provides the binding affinities of the proteins with Nifurtimox (DB11820). The last column ‘Protein name’ contains the names of the proteins corresponding to the PDB IDs, Supplementary Table S19c2: Comparison of binding affinities (kcal/mol) obtained from docking against Benznidazole (DB11989) with top 10 crystal structure and solved structure. This table has 3 columns: PDB ID; Binding score and Protein name. The first column ‘PDB ID’ provides the PDB IDs corresponding to the top 10 crystal structures of the *T. cruzi* proteins. The second column ‘Binding affinity (kcal/mol)’ provides the binding affinities of the proteins with Benznidazole (DB11989). The last column ‘Protein name’ contains the names of the proteins corresponding to the PDB IDs, Supplementary Table S20: The binding affinities of nifurtimox, aspirin, orlistat and benznidazole drugs with proteins involved in the pentose phosphate pathway (PPP). This table has 6 columns: PPP pathway proteins; PMID; DB11820 (Nifurtimox); DB00945 (Aspirin); DB1083 (Orlistat) and DB11989 (Benznidazole). The first column ‘PPP pathway proteins’ lists the proteins associated with PPP pathways. The second column ‘PMID’ provides the PMID from PubMed for the paper from where evidence of protein association with the PPP was collected. The third column ‘DB11820 (Nifurtimox)’ provides the binding energy of the Nifurtimox drug against the different proteins. The fourth column ‘DB00945 (Aspirin)’ provides the binding energy of Aspirin against the different proteins. The fifth column ‘DB01083 (Orlistat)’ provides the binding energy of Orlistat against the different proteins. The last column ‘DB11989 (Benznidazole)’ provides the binding energy of Benznidazole drug against the different proteins, Supplementary Table S21: The binding affinity of benznidazole with 19,523 human proteins. This table has 8 columns: Entry, Entry name, Status, Protein names, Gene names, Organism, Length, and DB11989. The first column ‘Entry’ list the protein IDs; the second column ‘Entry name’ list the name provided for each entry in the Alpha-fold database; the third column ‘Status’ provides the information on review status of each protein entry; the fourth column ‘Protein names’ list the names of each protein; the fifth column ‘Gene names’ list the name of the genes that encode the protein; the sixth column ‘Organism’ list the source organism of the protein; seventh column ‘Length’ list the length of each protein. The last column ‘DB11989’ lists benznidazole’s binding affinity against each protein, Supplementary Table S22: The binding affinity of 1516 FDA-approved drugs with the crystal structure of PGF (PDB: 2F38). This table has 2 columns: Ligand and Binding energy. The first column ‘Ligand’ list the drug IDs and second column ‘Binding Affinity’ list the binding energy of each drug against prostaglandin F synthase (2F38), Supplementary Table S23a: The binding affinity of *T. cruzi* solved protein structures (targets) with nifurtimox, aspirin, orlistat and benznidazole. This table has information in 5 columns: Protein ID, DB11820 (Nifurtimox), DB00945 (Aspirin), DB01083 (Orlistat) and DB11989 (Benznidazole). The first column ‘Protein ID’ provides the NCBI accession ID of the protein targets; The second column ‘DB11820 (Nifurtimox)’ provides the binding affinity (kcal/mol) of the protein target docked against Nifurtimox; The second column ‘DB00945 (Aspirin)’ provides the binding affinity (kcal/mol) of the protein target docked against Aspirin; The fourth column ‘DB01083 (Orlistat)’ provides the binding affinity (kcal/mol) of the protein target docked against Orlistat; The fifth column ‘DB11989 (Benznidazole)’ provides the binding affinity (kcal/mol) of the protein target docked against Benznidazole, Supplementray Table S23b1: The molecular docking statistics of each drug docked against 5004 *T. cruzi* CL Brener solved protein structures. This table has information in 2 columns for each drug: Metric and Values. The first column ‘Metric’ provides the name of the metric measured to know the overall results of the drug when docked against all the protein structures; The second column provides the measured value of each metric in kcal/mol, Supplementray Table S23b2: List of protein target with highest binding affinity for each drug. This table lists the protein target with the highest binding affinity out of the total 5004 *T. cruzi* CL Brener solved protein structures for each drug. The protein name, binding affinity, residure, NCBI protein accession ID, and NCBI gene accession ID is provided for each protein target, Supplementary Table S23c: The top five ranking *T. cruzi* protein targets for nifurtimox, aspirin, orlistat and benznidazole. This table has information in 3 columns for each drug: Protein ID, Score, and Protein name. The first column ‘Protein ID’ provides the NCBI accession ID of the protein targets; The second column ‘Score’ provides the binding affinity (kcal/mol) of the protein target when docked against the respective drug; The third column ‘Protein name’ provides the name of the top 5 protein targets for each drug, Supplementary Table S23d: Results of Welch’s T-Test applied to the binding affinity for different pairs of drug molecules. This table has information in 5 columns: Student T-Test, T-value, 95 percent confidence interval, Sample Estimates and Mean of the Differences. The first column ‘Student T-Test’ indicates the names of the drugs (drug pair) for which the binding affinities are tested; The second column ‘T-value’ provides the T-value on the basis of the binding affinities of the two drugs to the *T. cruzi* protein structures; The third column ‘95 percent confidence interval’ provides the 95 percent confidence interval on the basis of the binding affinities of the two drugs to the *T. cruzi* protein structures; The third column ‘Sample Estimates’ provides the sample estimates on the basis of the binding affinities of the two drugs to the *T. cruzi* protein structures, Supplementary Table S24a: The binding affinity of Plasmodium falciparum with Benznidazole (DB11989). This table has 3 columns. The first column ‘Protein ID of *P. falciparum* proteins’ provides the PDB ID of *P. falciparum* proteins; The second column ‘Binding affinity (kcal/mol)’ is the binding affinity of *P. falciparum* proteins when docked against Benznidazole (DB11989) drug; The third column ‘Name of the protein’ provides the name of the *P. falciparum* protein, Supplementary Table S24b: The binding affinity of Mycobacterium tuberculosis with Benznidazole (DB11989). This table has 3 columns. The first column ‘Protein ID of *M. tuberculosis* proteins’ provides the PDB ID of *M. tuberculosis* proteins; The second column ‘Binding affinity (kcal/mol)’ is the binding affinity of *M. tuberculosis* proteins when docked against Benznidazole (DB11989) drug; The third column ‘Name of the protein’ provides the name of the *M. tuberculosis* protein, Supplementary Table S24c: The binding affinity of Leishmania donovani with Benznidazole (DB11989). This table has 3 columns. The first column ‘Protein ID of Leishmania donovani proteins’ provides the PDB ID of L. donovani proteins; The second column ‘Binding affinity (kcal/mol)’ is the binding affinity of L. donovani proteins when docked against Benznidazole (DB11989) drug; The third column ‘Name of the protein’ provides the name of the L. donovani protein, Supplementary Table S24d: The binding affinity of Salmonella typhi with Benznidazole (DB11989). This table has 3 columns. The first column ‘Protein ID of Salmonella typhi proteins’ provides the PDB ID of S. typhi proteins; The second column ‘Binding affinity (kcal/mol)’ is the binding affinity of S. typhi proteins when docked against Benznidazole (DB11989) drug; The third column ‘Name of the protein’ provides the name of the S. typhi protein, Supplementary Table S24e: Results of Welch’s T-Test applied to the binding affinity of *T. cruzi* solved structures and other pathogens. This table has information in 4 columns: Student T-Test, T-value, 95 percent confidence interval, and Sample Estimates. The first column ‘Student T-Test’ indicates the name of the pathogen for which the binding affinities are tested against *T. cruzi* solved structures; The second column ‘T-value’ provides the T-value on the basis of the binding affinities of *T. cruzi* solved structures and the pathogen; The third column ‘95 percent confidence interval’ provides the 95 percent confidence interval on the basis of the binding affinities of *T. cruzi* solved structures and the pathogen; The fourth column ‘Sample Estimates’ provides the sample estimates on the basis of the binding affinities of *T. cruzi* solved structures and the pathogen, Supplementary Table S25: The binding affinity of FDA-approved drug molecules docked against Tc24. This table has information in 2 columns: Ligand, and Affinity (kcal/mol). The first column ‘Ligand’ provides the DrugBank ID of the drug molecules docked against Tc24; The second column ‘Affinity (kcal/mol)’ provides the strength of the interaction between the drug molecule and Tc24 that bind reversibly, Supplementary Table S26: Docking results of Benznidazole and Nifurtimox against Tc24 protein using AutoDock Vina. This table has information in 5 columns: Drug name, Mode (Top 30), Affinity (kcal/mol), Distance from the best mode (rmsd l.b.), and Distance from the best mode (rmsd u.b.). The first column ‘Drug name’ provides the name of the drug docked against Tc24; The second column Mode (Top 3) provides the orientation of the ligand relative to the receptor as well as the conformation of the ligand and receptor when bound to each other; The third column Affinity (kcal/mol) provides the strength of the interaction between the drug molecule and Tc24 that bind reversibly; The fourth and fifth columns ‘Distance from the best mode (rmsd l.b.)’ and ‘Distance from best mode (rmsd u.b.)’ provides the distance of that particular mode from the best mode in terms of Root Mean Square Deviation (RMSD)-upper bound and lower bound, Supplementary Table S27a: Simple parameters of *T. cruzi* pathways map determined using the network analyzer Cytoscape plugin. This table has information in 2 columns: Type and Statistics. The first column ‘Type’ provides the type of the parameter; The second column ‘Statistics’ provides the value of the parameter measured using Cytoscape, Supplementary Table S27b: Simple parameters of *T. cruzi* molecular map determined using the network analyzer Cytoscape plugin. This table has information in 2 columns: Type and Statistics. The first column ‘Type’ provides the type of the parameter; The second column ‘Statistics’ provides the value of the parameter measured using Cytoscape, Supplementary Table S27c: Simple parameters of *T. cruzi* drug map determined using the network analyzer Cytoscape plugin. This table has information in 2 columns: Type and Statistics. The first column ‘Type’ provides the type of the parameter; The second column ‘Statistics’ provides the value of the parameter measured using Cytoscape, Supplementary Table S28: List of *T. cruzi* genes (class: Trypanosomatidae) found from the TritrypDB database during Gene Ontology Analysis. This table has information in 7 columns: Gene ID, source_id, organism, Genomic Location (Gene), Description, Gene Type and Input ID. The first column ‘Gene ID’ provides the ID of the enriched gene; The second column ‘source_id’ provides the source ID of the enriched gene; The third column ‘Organism’ provides the name of the organism to which the enriched gene belongs; The fourth column ‘Gene Location (Gene)’ provides the location of the enriched gene; The fifth column ‘Description’ describes the enriched gene; The sixth column ‘Gene Type’ provides the type of the enriched gene; The seventh column ‘Input ID’ provides the input ID of the enriched gene, Supplementary Table S29: Total list of enriched genes that belong to *Trypanosoma cruzi* species (*Trypanosoma cruzi* CL Brener Esmeraldo-like, and *Trypanosoma cruzi* CL Brener Non-Esmeraldo-like). This table has information in 4 columns: Gene ID, source_id, organism, Genomic Location (Gene), Description, Gene Type, and Input ID. The first column ‘Gene ID’ provides the ID of the enriched gene; The second column ‘source_id’ provides the source ID of the enriched gene; The third column ‘Organism’ provides the name of the organism to which the enriched gene belongs; The fourth column ‘Gene Location (Gene)’ provides the location of the enriched gene; The fifth column ‘Description’ describes the enriched gene; The sixth column ‘Gene Type’ provides the type of the enriched gene; The seventh column ‘Input ID’ provides the input ID of the enriched gene, Supplementary Table S30a: List of enriched genes in Biological processes related to *Trypanosoma cruzi* CL Brener Non-Esmeraldo-like. This table has information in 11 columns: ID; Name; Bgd count; Result count; Result gene list; Pct of bgd; Fold enrichment; Odds ratio; *p*-value; Benjamini; and Bonferroni. The first column ‘ID’ provides the ID of the BP; The second column ‘Name’ provides the name of the BP; The third column ‘Bgd count’ provides the number of genes with this BP in the genome; The fourth column ‘Result count’ provides the number of genes with this BP in our analysis; The fifth ‘Result gene list’ provides the names of the genes with this BP in our analysis; The sixth column ‘Pct of Bgd’ provides percent of genes with this BP in our analysis divided by the percent of genes with this BP in the genome; The seventh column ‘Fold enrichment’ provides the percent that are present in your analysis of the genes in the genome with this BP; The eigth column ‘Odds ratio’ provides -The odds of the BP appearing in the gene list are the same as that for the background list; The ninth column ‘*p*-value’ provides the probability of seeing at least x number of genes out of the total n genes in the list annotated to the BP, given the proportion of genes in the whole genome that are annotated to that BP; The tenth column ‘Benjamini’ provides the Benjamini-Hochburg false discovery rate which is a method for controlling false discovery rates for type 1 errors; The eleventh column ‘Bonferroni’ provides the Bonferroni adjusted *p*-values which is a method for correcting significance based on multiple comparisons, Supplementary Table S30b: List of enriched genes in molecular function related to *Trypanosoma cruzi* CL Brener Non-Esmeraldo-like. This table has information in 11 columns: ID; Name; Bgd count; Result count; Result gene list; Pct of bgd; Fold enrichment; Odds ratio; *p*-value; Benjamini; and Bonferroni. The first column ‘ID’ provides the ID of the MF; The second column ‘Name’ provides the name of the MF; The third column ‘Bgd count’ provides the number of genes with this MF in the genome; The fourth column ‘Result count’ provides the number of genes with this MF in our analysis; The fifth ‘Result gene list’ provides the names of the genes with this MF in our analysis; The sixth column ‘Pct of Bgd’ provides percent of genes with this MF in our analysis divided by the percent of genes with this MF in the genome; The seventh column ‘Fold enrichment’ provides the percent that are present in your analysis of the genes in the genome with this MF; The eigth column ‘Odds ratio’ provides -The odds of the MF appearing in the gene list are the same as that for the background list; The ninth column ‘*p*-value’ provides the probability of seeing at least x number of genes out of the total n genes in the list annotated to the MF, given the proportion of genes in the whole genome that are annotated to that MF; The tenth column ‘Benjamini’ provides the Benjamini-Hochburg false discovery rate which is a method for controlling false discovery rates for type 1 errors; The eleventh column ‘Bonferroni’ provides the Bonferroni adjusted *p*-values which is a method for correcting significance based on multiple comparisons, Supplementary Table S30c: List of enriched genes in cellular component related to *Trypanosoma cruzi* CL Brener Non-Esmeraldo-like. This table has information in 11 columns: ID; Name; Bgd count; Result count; Result gene list; Pct of bgd; Fold enrichment; Odds ratio; *p*-value; Benjamini; and Bonferroni. The first column ‘ID’ provides the ID of the CC; The second column ‘Name’ provides the name of the CC; The third column ‘Bgd count’ provides the number of genes with this CC in the genome; The fourth column ‘Result count’ provides the number of genes with this CC in our analysis; The fifth ‘Result gene list’ provides the names of the genes with this CC in our analysis; The sixth column ‘Pct of Bgd’ provides percent of genes with this CC in our analysis divided by the percent of genes with this CC in the genome; The seventh column ‘Fold enrichment’ provides the percent that are present in your analysis of the genes in the genome with this CC; The eigth column ‘Odds ratio’ provides -The odds of the CC appearing in the gene list are the same as that for the background list; The ninth column ‘*p*-value’ provides the probability of seeing at least x number of genes out of the total n genes in the list annotated to the CC, given the proportion of genes in the whole genome that are annotated to that CC; The tenth column ‘Benjamini’ provides the Benjamini-Hochburg false discovery rate which is a method for controlling false discovery rates for type 1 errors; The eleventh column ‘Bonferroni’ provides the Bonferroni adjusted *p*-values which is a method for correcting significance based on multiple comparisons, Supplementary Table S30d: List of enriched genes in metabolic pathways related to *Trypanosoma cruzi* CL Brener Non-Esmeraldo-like. This table has information in 11 columns: ID; Name; Bgd count; Result count; Result gene list; Pct of bgd; Fold enrichment; Odds ratio; *p*-value; Benjamini; and Bonferroni. The first column ‘ID’ provides the ID of the MP; The second column ‘Name’ provides the name of the MP; The third column ‘Bgd count’ provides the number of genes with this MP in the genome; The fourth column ‘Result count’ provides the number of genes with this MP in our analysis; The fifth ‘Result gene list’ provides the names of the genes with this MP in our analysis; The sixth column ‘Pct of Bgd’ provides percent of genes with this MP in our analysis divided by the percent of genes with this MP in the genome; The seventh column ‘Fold enrichment’ provides the percent that are present in your analysis of the genes in the genome with this MP; The eigth column ‘Odds ratio’ provides -The odds of the MP appearing in the gene list are the same as that for the background list; The ninth column ‘*p*-value’ provides the probability of seeing at least x number of genes out of the total n genes in the list annotated to the MP, given the proportion of genes in the whole genome that are annotated to that MP; The tenth column ‘Benjamini’ provides the Benjamini-Hochburg false discovery rate which is a method for controlling false discovery rates for type 1 errors; The eleventh column ‘Bonferroni’ provides the Bonferroni adjusted *p*-values which is a method for correcting significance based on multiple comparisons, Suppelementary Table S31a: List of enriched genes in biological process (BP) related to *Trypanosoma cruzi* CL Brener Esmeraldo-like. This table has information in 11 columns: ID; Name; Bgd count; Result count; Result gene list; Pct of bgd; Fold enrichment; Odds ratio; *p*-value; Benjamini; and Bonferroni. The first column ‘ID’ provides the ID of the BP; The second column ‘Name’ provides the name of the BP; The third column ‘Bgd count’ provides the number of genes with this BP in the genome; The fourth column ‘Result count’ provides the number of genes with this BP in our analysis; The fifth ‘Result gene list’ provides the names of the genes with this BP in our analysis; The sixth column ‘Pct of Bgd’ provides percent of genes with this BP in our analysis divided by the percent of genes with this BP in the genome; The seventh column ‘Fold enrichment’ provides the percent that are present in your analysis of the genes in the genome with this BP; The eigth column ‘Odds ratio’ provides -The odds of the BP appearing in the gene list are the same as that for the background list; The ninth column ‘*p*-value’ provides the probability of seeing at least x number of genes out of the total n genes in the list annotated to the BP, given the proportion of genes in the whole genome that are annotated to that BP; The tenth column ‘Benjamini’ provides the Benjamini-Hochburg false discovery rate which is a method for controlling false discovery rates for type 1 errors; The eleventh column ‘Bonferroni’ provides the Bonferroni adjusted *p*-values which is a method for correcting significance based on multiple comparisons, Supplementary Table S31b: List of enriched genes in molecular function (MF) related to *Trypanosoma cruzi* CL Brener Esmeraldo-like. This table has information in 11 columns: ID; Name; Bgd count; Result count; Result gene list; Pct of bgd; Fold enrichment; Odds ratio; *p*-value; Benjamini; and Bonferroni. The first column ‘ID’ provides the ID of the MF; The second column ‘Name’ provides the name of the MF; The third column ‘Bgd count’ provides the number of genes with this MF in the genome; The fourth column ‘Result count’ provides the number of genes with this MF in our analysis; The fifth ‘Result gene list’ provides the names of the genes with this MF in our analysis; The sixth column ‘Pct of Bgd’ provides percent of genes with this MF in our analysis divided by the percent of genes with this MF in the genome; The seventh column ‘Fold enrichment’ provides the percent that are present in your analysis of the genes in the genome with this MF; The eigth column ‘Odds ratio’ provides -The odds of the MF appearing in the gene list are the same as that for the background list; The ninth column ‘*p*-value’ provides the probability of seeing at least x number of genes out of the total n genes in the list annotated to the MF, given the proportion of genes in the whole genome that are annotated to that MF; The tenth column ‘Benjamini’ provides the Benjamini-Hochburg false discovery rate which is a method for controlling false discovery rates for type 1 errors; The eleventh column ‘Bonferroni’ provides the Bonferroni adjusted *p*-values which is a method for correcting significance based on multiple comparisons, Supplementary Table S31c: List of enriched genes in cellular component related to *Trypanosoma cruzi* CL Brener Esmeraldo-like. This table has information in 11 columns: ID; Name; Bgd count; Result count; Result gene list; Pct of bgd; Fold enrichment; Odds ratio; *p*-value; Benjamini; and Bonferroni. The first column ‘ID’ provides the ID of the CC; The second column ‘Name’ provides the name of the CC; The third column ‘Bgd count’ provides the number of genes with this CC in the genome; The fourth column ‘Result count’ provides the number of genes with this CC in our analysis; The fifth ‘Result gene list’ provides the names of the genes with this CC in our analysis; The sixth column ‘Pct of Bgd’ provides percent of genes with this CC in our analysis divided by the percent of genes with this CC in the genome; The seventh column ‘Fold enrichment’ provides the percent that are present in your analysis of the genes in the genome with this CC; The eigth column ‘Odds ratio’ provides -The odds of the CC appearing in the gene list are the same as that for the background list; The ninth column ‘*p*-value’ provides the probability of seeing at least x number of genes out of the total n genes in the list annotated to the CC, given the proportion of genes in the whole genome that are annotated to that CC; The tenth column ‘Benjamini’ provides the Benjamini-Hochburg false discovery rate which is a method for controlling false discovery rates for type 1 errors; The eleventh column ‘Bonferroni’ provides the Bonferroni adjusted *p*-values which is a method for correcting significance based on multiple comparisons, Supplementary Table S31d: List of enriched genes in molecular function related to *Trypanosoma cruzi* CL Brener Esmeraldo-like. This table has information in 11 columns: ID; Name; Bgd count; Result count; Result gene list; Pct of bgd; Fold enrichment; Odds ratio; *p*-value; Benjamini; and Bonferroni. The first column ‘ID’ provides the ID of the MP; The second column ‘Name’ provides the name of the MP; The third column ‘Bgd count’ provides the number of genes with this MP in the genome; The fourth column ‘Result count’ provides the number of genes with this MP in our analysis; The fifth ‘Result gene list’ provides the names of the genes with this MP in our analysis; The sixth column ‘Pct of Bgd’ provides percent of genes with this MP in our analysis divided by the percent of genes with this MP in the genome; The seventh column ‘Fold enrichment’ provides the percent that are present in your analysis of the genes in the genome with this MP; The eigth column ‘Odds ratio’ provides -The odds of the MP appearing in the gene list are the same as that for the background list; The ninth column ‘*p*-value’ provides the probability of seeing at least x number of genes out of the total n genes in the list annotated to the MP, given the proportion of genes in the whole genome that are annotated to that MP; The tenth column ‘Benjamini’ provides the Benjamini-Hochburg false discovery rate which is a method for controlling false discovery rates for type 1 errors; The eleventh column ‘Bonferroni’ provides the Bonferroni adjusted *p*-values which is a method for correcting significance based on multiple comparisons, Supplementary file S1: The 86,990 abstracts (Google Scholar-85,600, PubMed-1390), as of February 2021 were collected using the literature mining approach, Supplementary file S2: The 103,868 abstracts (Google Scholar-96,300, PubMed-7568), as of February 2021, were screened to obtain the molecular information related to *T. cruzi*, Supplementary file S3: The 1693 abstracts with the terms “*T. cruzi*” AND “drug” were downloaded from Google Scholar and PubMed.
