# Supplementary material for: A Data-Driven Approach to Construct a Molecular Map of Trypanosoma cruzi to Identify Drugs and Vaccine Targets
Source: Vaccines (Basel). 2023 Jan 26;11(2):267. doi: 10.3390/vaccines11020267 (PMC9963959; doi:10.3390/vaccines11020267)
Supplement: Supplementary file 1 [file vaccines-11-00267-s001.zip › Supplementary Figures/Supplementary Figure Descriptions.pdf]

A data-driven approach to construct a molecular map of *Trypanosoma cruzi* to identify drug and vaccine targets

Swarsat Kaushik Nath[1#], Preeti P.[1], Trapti Sharma[1], Priya Kumari[1], Sweety Shinde[1], Nikita Garg[1], Kartavya Mathur[1], Nevidita Arambam[1], Divyank Harjani[1], Manpriya Raj, Garwit Kwatra[1], Sayantan Venkatesh[1], Alakto Choudhoury[1], Saima Bano[1], Prashansa Tayal[1], Mahek Sharan[1], Ruchika Arora[1], Ulrich Strych[2], Peter J. Hotez[2, 3], Maria Elena Bottazzi[2, 3] and Kamal Rawal[1*#]

1 Amity Institute of Biotechnology, Amity University Uttar Pradesh, India.
2 Texas Children's Hospital Center for Vaccine Development, Departments of Pediatrics and Molecular Virology and Microbiology, National School of Tropical Medicine, Baylor College of Medicine, Houston, TX, USA.
3 Department of Biology, Baylor University, Waco, Texas, USA.

*Corresponding Author:
Dr. Kamal Rawal
Centre for Computational Biology and Bioinformatics, AIB
Amity University, Noida, Uttar Pradesh, India.
Email address: kamal.rawal@gmail.com

#These authors have contributed equally.

Website: https://tinyurl.com/Tcruzipathwaymapx

S. No.	Supplementary Figure no.	Description	Link
1.	**Supplementary Figure 1**	**Supplementary Figure 1: The schematic diagram describing the flowchart to achieve the methodology.**	https://tinyurl.com/suppfig1
2.	**Supplementary Figure 2**	**Supplementary Figure 2: The standard notations for the graphical representation of species and edges in cell designer v.4.4.2.**	https://tinyurl.com/suppfig2
3.	**Supplementary Figure 3**	**Supplementary Figure 3: Docking of Tc24 protein with Benznidazole and Nifurtimox using AutoDock Vina. (a.** Benznidazole, **b.** Nifurtimox). The top scoring pose of docking affinity against Tc24 for Benznidazole is -6.9 kcal/mol while the docking affinity for Nifurtimox is -6.7 kcal/mol.	https://tinyurl.com/suppfig3
4.	**Supplementary Figure 4**	**Supplementary Figure 4A: *Trypanosoma cruzi* CL Brener Esmeraldo-like.** Enriched GO terms of genes (**a.** Biological process, **b.** Cellular component, **c.** Molecular function). The size of the dots and their color gradient (according to the legend) reflects the log10 value of the p value of each GO term, larger dots represent higher significance (all p values <0.05). X and Y axes represent semantic spaces which have no intrinsic meaning. REVIGO uses multi-dimensional scaling to reduce the dimensionality of a matrix of the GO terms' pairwise semantic similarities. This results in semantically similar GO terms remaining close together in the plot.	https://tinyurl.com/suppfig4
		**Supplementary Figure 4B: *Trypanosoma cruzi* CL Brener Non-Esmeraldo-like.** Enriched GO terms of genes (**a.** Biological process, **b.** Cellular component, **c.** Molecular function). The size of the dots and their color gradient (according to the legend) reflects the log10 value of the p value of each GO term, larger dots represent higher significance (all p values <0.05). X and Y axes represent semantic spaces which have no intrinsic meaning. REVIGO uses multi-dimensional scaling to reduce the dimensionality of a matrix of the GO terms' pairwise semantic similarities. This results in semantically similar GO terms remaining close together in the plot.	
5.	**Supplementary Figure 5**	**Supplementary Figure 5A: *Trypanosoma cruzi* CL Brener Non-Esmeraldo-like.** "Interactive graph" view of REVIGO (**a.** Biological process, **b.** Cellular component, **c.** Molecular function). Bubble color indicates the user-provided p-value. Highly similar GO terms are linked by	https://tinyurl.com/suppfig5

		edges in the graph, where the line width indicates the degree of similarity.	
		**Supplementary Figure 5B: *Trypanosoma cruzi* CL Brener Esmeraldo-like.** "Interactive graph" view of REVIGO (**a.** Biological process, **b.** Cellular component, **c.** Molecular function). Bubble color indicates the user-provided p-value. Highly similar GO terms are linked by edges in the graph, where the line width indicates the degree of similarity.	
6.	**Supplementary Figure 6**	**Supplementary Figure 6:** Gene ontology (GO) classification, KEGG/MetaCyc analysis of the *T. cruzi* genes **(a.)** *T. cruzi* CL Brener Esmeraldo like **(b.)***T. cruzi* CL Brener Non-Esmeraldo like). The percentage regulated genes based on GO annotations and KEGG/MetaCyc pathways. **(c)** word cloud graph depicting enriched Metabolic Pathway **(1)** *Trypanosoma cruzi* CL Brener Esmeraldo-lik*e* **(2)** *Trypanosoma cruzi CL* Brener Non-Esmeraldo-like.	https://tinyurl.com/suppfig6

* % regulated genes are the percentage of the background or GO/pathway-related genes.
