# Supplementary material for: A Data-Driven Approach to Construct a Molecular Map of Trypanosoma cruzi to Identify Drugs and Vaccine Targets
Source: Vaccines (Basel). 2023 Jan 26;11(2):267. doi: 10.3390/vaccines11020267 (PMC9963959; doi:10.3390/vaccines11020267)
Supplement: Supplementary file 1 [file vaccines-11-00267-s001.zip › Supplementary Tables/Supplementary Table 27.pdf]

Supplementary Table 27 a: Standard parameters of *T.cruzi* pathways map determined using the network analyzer Cytoscape plugin. This table has information in 2 columns: Type and Statistics. The first column 'Type' provides the type of the parameter; The second column 'Statistics' provides the value of the parameter measured using Cytoscape.

S.No.	Type	Statistics
1	Number of nodes	1929
2	Number of edges	2088
3	Average number of neighbors	2.183
4	Network Diameter	28
5	Network Radius	14
6	Characteristics path length	11.735
7	Clustering coefficient	0.000
8	Network density	0.014
9	Connected components	0.728
10	Multi-edge node pair	0.085
11	Number of self-loops	192
12	Analysis time (sec.)	0.060

Supplementary Table 27 b: Standard parameters of *T.cruzi* molecular map determined using the network analyzer Cytoscape plugin. This table has information in 2 columns: Type and Statistics. The first column 'Type' provides the type of the parameter; The second column 'Statistics' provides the value of the parameter measured using Cytoscape.

S.No.	Type	Statistics
1	Number of nodes	4016
2	Number of edges	3468
3	Average number of neighbors	2.024
4	Network Diameter	22
5	Network Radius	11
6	Characteristics path length	8.332
7	Clustering coefficient	0.000
8	Network density	0.025
9	Connected components	0.556
10	Multi-edge node pair	0.101
11	Number of self-loops	812
12	Analysis time (sec.)	0.133

Supplementary Table 27 c: Standard parameters of *T.cruzi* drug map determined using the network analyzer Cytoscape plugin. This table has information in 2 columns: Type and Statistics. The first column 'Type' provides the type of the parameter; The second column 'Statistics' provides the value of the parameter measured using Cytoscape.

S.No.	Type	Statistics
1	Number of nodes	51
2	Number of edges	52
3	Average number of neighbors	2.039
4	Network Diameter	18
5	Network Radius	9
6	Characteristics path length	6.915
7	Clustering coefficient	0.000
8	Network density	0.041
9	Connected components	0.412
10	Multi-edge node pair	0.062
11	Number of self loops	1
12	Analysis time (sec.)	0.019
