# Supplementary material for: A Data-Driven Approach to Construct a Molecular Map of Trypanosoma cruzi to Identify Drugs and Vaccine Targets
Source: Vaccines (Basel). 2023 Jan 26;11(2):267. doi: 10.3390/vaccines11020267 (PMC9963959; doi:10.3390/vaccines11020267)
Supplement: Supplementary file 1 [file vaccines-11-00267-s001.zip › Supplementary Tables/Supplementary Table Index.pdf]

S. No.	TITLE	LEGEND	LINK
1	Supplementary Table 1	**Supplementary Table 1a: List of all the curated evidence for the construction of *T. cruzi* map from 46 pathways.** This table provides information about 46 pathways derived from literature; constituent molecules and the literature evidence. The information is presented in 4 columns: Name; Molecule; Evidence and PMID/LINK. The first column 'Name' provides information about the name of pathways reported in the literature; The second column 'Molecule' lists the specific molecule involved in the given pathway. The third column 'Evidence' shows specific lines/statements/paragraphs from the article/research paper that provides evidence for the role of the molecule in the specific pathway. The last column 'PMID/LINK' provides PMID or hyperlink of the paper from where evidence has been collected.	Supplementary Table 1.xlsx
		**Supplementary Table 1 b: List of curated evidence of different biological molecule interactions for the construction of 46 *T. cruzi* pathway map.** This table shows a curated list of biological molecule interactions evident to construct the 46 *T. cruzi* pathway map. [Molecule 1 = first molecule of the interaction; Molecule 2 = second molecule of the interaction; Evidence = Statement from the article that provides evidence for the role of the molecule in the specific pathway; Interactions = type of interaction between molecule 1 and molecule 2; PMID/LINK = PMID or link of the paper from where evidence has been collected].	
2	Supplementary Table 2	**Supplementary Table 2 a: List of curated pathways and their classifications.** This table shows the classification of curated pathways and the sources from where they were extracted. This table has information in 4 columns: Name; Classification; Source 1, and Source 2. The first column 'Name' provides information about the name of pathways reported in the literature about *T. cruzi*; The second column 'Classification' lists the classes of pathways. The third column 'Source 1' is the first source of information from where pathways are extracted. The last column 'Source 2' is the second source of information from where pathway classifications are extracted.	Supplementary Table 2.xlsx
		**Supplementary Table 2 b: List of curated pathways and their classifications.** This table holds the list of curated pathways and their classifications based on the organism and host interaction. [Pathway name = Name of the pathway; Pathway from	

		Organism = Source organism of the pathway; Type of pathway = The type of a specific pathway].	
3	Supplementary Table 3	**Supplementary Table 3: The list of *Tc*-CLB genes retrieved from the NCBI database.** This table has information in 8 columns: Tax_id, Organism name, GeneID, CurrentID, Status, Symbol, Aliases, and Description. The first column 'Tax_id' is the taxonomic ID of *T. cruzi*; The second column 'Organism name' is the name of the organism with strain detail. The third column 'GeneID' is the specific ID provided to the gene by NCBI. The fourth column 'CurrentID' is the ID provided by NCBI. 'Status' gives the current status of the gene in NCBI. 'Symbol' is the gene symbol. 'Aliases' gives the available aliases of gene symbols. The last column 'Description' provides the name of the gene.	Supplementary Table 3.xlsx
4	Supplementary Table 4	**Supplementary Table 4: The list of unique *Tc*-CLB genes used for the construction of the comprehensive molecular map.**	Supplementary Table 4.xlsx
5	Supplementary Table 5	**Supplementary Table 5 a: The List of curated molecular evidence of *T. cruzi* genes with their interactions.** This table has information in 5 columns: S.No., Molecule Layer 1 (*T. cruzi*), Molecule layer 2 (Human/Mice), Evidence, and PMID/LINK. The first column 'S.No.' is the serial number; The second column 'Molecule layer 1 (*T.cruzi*)' is the first molecule from *T. cruzi* in the interaction. The third column 'Molecule layer 2 (Human/Mice)' is the second molecule from Humans or Mice in the interaction. The fourth column is 'Evidence' which shows specific lines/statements/paragraphs from the article/research paper that provides evidence for the role of the molecule in the specific pathway. The last column 'PMID/LINK' provides a PMID or hyperlink of the paper from where evidence has been collected.	Supplementary Table 5.xlsx
		**Supplementary Table 5 b: The Summary of the source of the information.** [Name = Name of the organism whose information is collected; Keyword = phrases used for literature search; PubMed hits = Number of hits found in Pubmed; Google scholar hits; Number of hits found in Google Scholar; Date = till what date data is collected].	
6	Supplementary Table 6	**Supplementary Table 6: The list of *Tc*-CLB proteins retrieved from the Uniprot database.** This table has information in 4 columns: Entry, Protein names, Gene names, and Organism. The first column 'Entry' provides UniProt ID for a specific protein entry; The second column 'Protein names' lists the names of the proteins. The third column 'Gene names' provides	Supplementary Table 6.xlsx

		the names of the genes that encode the protein. The last column 'Organism' provides the name of the organism from which the protein was extracted.	
7	Supplementary Table 7	**Supplementary Table 7: The list of unique *T. cruzi* proteins used for the construction of the comprehensive molecular map [n=3109].**	Supplementary Table 7.xlsx
8	Supplementary Table 8	**Supplementary Table 8 a: The list of curated molecular evidence of *T. cruzi* proteins with their interactions.** This table has information in 4 columns: Protein, Molecule 2 (Human/Mice), Evidence, and PMID/Link. The first column 'Protein' provides the name of the protein from *T.cruzi*; The second column 'Molecule2 (Human/Mice)' lists the second molecule from Humans or Mice in the interaction. The third column 'Evidence' shows specific lines/statements/paragraphs from the article/research paper that provides evidence for the role of the molecule in the specific pathway. The last column 'PMID/LINK' provides a PMID or hyperlink of the paper from where evidence has been collected.<br><br>**Supplementary Table 8 b: The Summary of the source of the information.** [Name = Name of the organism whose information is collected; Keyword = phrases used for literature search; PubMed hits = Number of hits found in Pubmed; Google scholar hits; Number of hits found in Google Scholar; Date = till what date data is collected].	Supplementary Table 8.xlsx
9	Supplementary Table 9	**Supplementary Table 9: The list of nodes involved in the comprehensive molecular map of *T. cruzi.*** This table has information in 12 columns: class, id, name, compartment, positionToCompartment, included, quantity type, initialQuantity, substanceUnits, hasOnlySubstanceUnits, b.c., and constants. The first column 'class' provides molecule type; The second column 'id' is the specific ID assigned to each entry. The third column 'name' lists the name of the molecule. 'compartment' is the compartment where the protein belongs; 'positionToCompartment' provides information on where the molecule is located. 'included' provides information on interactions. 'quantity' provides selected option for quantity an entry in terms of either amount as molecular/item count or concentration as units of substance/units of size; 'initialQuanity' list the value set as initial quantity to run the simulation; 'substanceUnit' provides the unit assigned to each entry. 'hasOnlySubstance' gives a Boolean (True or False) on if species quantity always be as substance or substance/size; 'b.c.' is the boundary condition, which is a boolean (True or False) on should a rate of	Supplementary Table 9.xlsx

		change equation be constructed for the species based on the system of reactions. The last column 'constants' is a boolean (True or False) on if the species quantity is constant.	
10	Supplementary Table 10	**Supplementary Table 10: List of edges involved in the comprehensive molecular map of *T. cruzi*.** This table has 7 columns: type; id; reversible; fast; reactants; products and modifiers. The first column 'type' provides information on the different types/categories of the reactions involved in the molecular map. The second column 'id' is the specific ID assigned to each entry. The third column 'reversible' gives a Boolean (True or False) on if the reaction is reversible or not. The fourth column 'fast' provides a Boolean (True or False) on if the reaction is fast or not. The fifth column 'reactants' lists unique molecule IDs (from Supplementary Table 9) of the reactant molecules in the reaction. The sixth column 'products' lists unique molecule IDs (from Supplementary Table 9) of the product formed in the reaction. The last column 'modifiers' lists unique molecule IDs (from Supplementary Table 9) of molecules that act as modifiers in the reaction, if any.	Supplementary Table 10.xlsx
11	Supplementary Table 11	**Supplementary Table 11: The list of the total number of curated abstracts retrieved from Google scholar and PubMed against 46 unique *T. cruzi* pathways.** This table has 5 columns: Name; Keyword Used; Google scholar hits; PubMed hits and Date. The first column 'Name' provides the list of *T. cruzi* pathways collected from literature and used for the construction of the molecular map. The second column 'Keyword Used' provides information about the key terms that were used for the literature search. The third column 'Google scholar hits' contains the number of abstracts/hits obtained from Google Scholar using the respective keyword. The fourth column 'PubMed hits' contains the number of abstracts/hits obtained from PubMed using the respective keyword. The last column 'Date' list the date till when the literature was extracted.	Supplementary Table 11.xlsx
12	Supplementary Table 12	**Supplementary Table 12: The list of nodes involved in the network of *T. cruzi* pathway.** This table has information in 10 columns: class, id, name, compartment, positionToCompartment, quantity type, initialQuantity, hasOnlySubstanceUnits, b.c., and constants. The first column 'class' provides molecule type; The second column 'id' is the specific ID assigned to each entry. The third column 'name' lists the name of the molecule. 'compartment' is the compartment where the molecule belongs; 'positionToCompartment' provides information on	Supplementary Table 12.xlsx

		where the molecule is located. 'quantity type' provides a selected option for the quantity of an entry in terms of either amount as molecular/item count or concentration as units of substance/units of size; 'initialQuanity' list the value set as initial quantity to run the simulation; 'hasOnlySubstance' gives a Boolean (True or False) on if species quantity always be as substance or substance/size; 'b.c.' is the boundary condition, which is a boolean (True or False) on should a rate of change equation be constructed for the species based on the system of reactions. The last column 'constants' is a boolean (True or False) on if the species quantity is constant.	
13	Supplementary Table 13	**Supplementary Table 13: List of edges involved in the network of *T. cruzi* pathways.** This table has 7 columns: type; id; reversible; fast; reactants; products and modifiers. The first column 'type' provides information on the different types/categories of the reactions involved in the molecular map. The second column 'id' is the specific ID assigned to each entry. The third column 'reversible' gives a Boolean (True or False) on if the reaction is reversible or not. The fourth column 'fast' provides a Boolean (True or False) on if the reaction is fast or not. The fifth column 'reactants' lists unique molecule IDs (from Supplementary Table 9) of the reactant molecules in the reaction. The sixth column 'products' lists unique molecule IDs (from Supplementary Table 9) of the product formed in the reaction. The seventh column 'modifiers' lists unique molecule IDs (from Supplementary Table 9) of molecules that act as modifiers in the reaction, if any.	Supplementary Table 13.xlsx
14	Supplementary Table 14	**Supplementary Table 14: The docking of proteins from the Ubiquitin Proteasome pathway with Nifurtimox and Benznidazole.** This table has 3 columns: Protein name, DB11820 (Nifurtimox) and DB11989 (Benznidazole). The first column 'Protein name' contains the list of proteins present in the ubiquitin-proteasome pathway which were docked against Nifurtimox and Benznidazole. The second column 'DB11820 (Nifurtimox)' provides the binding affinities of the various pathway proteins when docked against Nifurtimox. The third column 'DB11989 (Benznidazole)' provides the binding affinities of the various proteins when docked against Benznidazole.	Supplementary Table 14.xlsx
15	Supplementary Table 15	**Supplementary Table 15 a: List of curated molecular evidence of *T. cruzi* drugs with its interactions.** This table has 6 columns: Name; Keyword; PubMed hits; Google scholar hits; Date; and Total. The first column 'Name' provides information on the name of the molecule. The second column 'Keyword' provides information about the key terms that were used for the literature search. The third	Supplementary Table 15.xlsx

		column 'PubMed hits' contains the number of abstracts obtained by using PubMed. The fourth column 'Google Scholar hits' contains the number of abstracts obtained by Google Scholar. The fifth column 'Date' provides information about the date on which the literature search was performed/completed. The sixth column 'Total' provides the sum total of hits from PubMed and Google Scholar combined.<br><br>**Supplementary Table 15 b: List of curated molecular evidence of *T.cruzi* drugs with its interaction.** This table has 4 columns: DRUG; EVIDENCE; LINK/PMID and DATE. The first column 'DRUG' provides the name of the drug. The second column 'EVIDENCE' shows specific lines/statements/paragraphs from the article/research paper that provides evidence of the interaction between the drug and *T. cruzi* molecules. The third column 'LINK/PMID' provides PMID of the paper from where the evidence has been collected. The fourth column 'Date' provides information about the date on which the literature search was performed/completed.<br><br>**Supplementary Table 15 c: List of curated molecular evidence of *T.cruzi* drugs with its interaction.** This table has 5 columns: DRUG, MOLECULE, EVIDENCE; LINK and DATE. The first column 'DRUG' provides a list of the drug names. The second column 'MOLECULE' provides the name of the molecule that interacts with the drug; The third column 'EVIDENCE' shows specific lines/statements/paragraphs from the article/research paper that provides evidence of the interaction between drugs and molecules. The fourth column 'LINK' provides a hyperlink of the paper from where the evidence has been collected. The fifth column 'Date' provides information about the date on which the literature search was performed/completed.	
16	Supplementary Table 16	**Supplementary Table 16: List of nodes involved in the comprehensive molecular map of drugs.** This table has 10 columns: class; id; name; compartment; positionToCompartment; quantity type; initialQuantity; hasOnlySubstanceUnits; b.c. and constants. The first column 'class' provides information on the various categories of nodes present in the network i.e., drug, protein, etc. The second column 'id' is the specific ID assigned to each entry. The third column 'name' contains the names of the different molecules. The fourth column 'compartment' is the compartment where the molecule belongs. The fifth column 'positionToCompartment' provides information on	Supplementary Table 16.xlsx

		where the molecule is located. The sixth column 'quantity type' provides a selected option for the quantity of an entry in terms of either amount as molecular/item count or concentration as units of substance/units of size. The seventh column 'initialQuantity' contains the value set as initial quantity to run the simulation. The eighth column 'hasOnlySubstanceUnits' gives a Boolean (True or False) on if species quantity always be as substance or substance/size. The ninth column 'b.c.' is the boundary condition, which is a boolean (True or False) on should a rate of change equation be constructed for the species based on the system of reactions. The last column 'constants' is a boolean (True or False) on if the species quantity is constant.	
17	Supplementary Table 17	**Supplementary Table 17: List of edges involved in the comprehensive molecular map of drugs.** This table has 6 columns: type; id; reversible; fast; reactants and products. The first column 'type' provides information on the type of reactions between reactants i.e., state transitions or influences. The second column 'id' is the specific ID assigned to each entry. The third column 'reversible' helps us to know whether the reaction involved is reversible or not. The fourth column 'fast' provides information about the speed of the reaction, whether it's fast or slow. The fifth column 'reactants' contains the IDs given to the reactant(s) involved. The last column 'products' contains the IDs given to the product(s) involved.	Supplementary Table 17.xlsx
18	Supplementary Table 18	**Supplementary Table 18: The list of drugs used in the network with its experimental status.** This table has 5 columns: Drug Name; PMID/link; Experimental status of the drug; Drug Bank ID and FDA-approved. The first column 'Drug Name' provides the list of various *T. cruzi* drugs used in the network and which are in different stages of development; The second column 'PMID/link' contains the link for the paper from which the data was collected; The third column 'Experimental status of the drug' provides the experimental status of the drug; The fourth column 'DrugBank ID' contains the IDs of the drugs that can be used to access the details of the drugs from the DrugBank Database; The last column 'FDA Approved' provides information on the developmental stage of the drugs i.e., approved, investigational, etc.	Supplementary Table 18.xlsx
19	Supplementary Table 19	**Supplementary Table 19 a: The binding affinity of the crystal structure of *T. cruzi* docked with Nifurtimox (DB11820).** This table has 3 columns: PDB ID; Binding affinity and Protein name. The first column 'PDB ID' provides the PDB IDs corresponding to the available crystal structures of the 127 proteins of *T. cruzi*; The second column 'Binding affinity	Supplementary Table 19.xlsx

		(kcal/mol)' provides the binding affinities of the 127 *T. cruzi* proteins with Nifurtimox; The third column 'Protein name' contains the names of the proteins corresponding to the PDB IDs.	
		**Supplementary Table 19 b: The binding affinity of the crystal structure of *T. cruzi* docked with Benznidazole (DB11989).** This table has 3 columns: PDB ID; Binding affinity and Protein name. The first column 'PDB ID' provides the PDB IDs corresponding to the available crystal structures of the 127 proteins of *T. cruzi*; The second column 'Binding affinity (kcal/mol)' provides the binding affinities of the 127 *T. cruzi* proteins with Benznidazole; The third column 'Protein name' contains the names of the proteins corresponding to the PDB IDs.	
		**Supplementary Table 19 c1: Comparison of binding affinities (kcal/mol) obtained from docking against Nifurtimox (DB11820) with top 10 crystal structure and solved structure.** This table has 3 columns: PDB ID; Binding score and Protein name. The first column 'PDB ID' provides the PDB IDs corresponding to the top 10 crystal structures of the *T. cruzi* proteins. The second column 'Binding affinity (kcal/mol)' provides the binding affinities of the proteins with Nifurtimox (DB11820). The last column 'Protein name' contains the names of the proteins corresponding to the PDB IDs.	
		**Supplementary Table 19 c2: Comparison of binding affinities (kcal/mol) obtained from docking against Benznidazole (DB11989) with top 10 crystal structure and solved structure.** This table has 3 columns: PDB ID; Binding score and Protein name. The first column 'PDB ID' provides the PDB IDs corresponding to the top 10 crystal structures of the *T. cruzi* proteins. The second column 'Binding affinity (kcal/mol)' provides the binding affinities of the proteins with Benznidazole (DB11989). The last column 'Protein name' contains the names of the proteins corresponding to the PDB IDs.	
20	Supplementary Table 20	**Supplementary table 20: The binding affinities of nifurtimox, aspirin, orlistat and benznidazole drugs with proteins involved in the pentose phosphate pathway (PPP).** This table has 6 columns: PPP pathway proteins; PMID; DB11820 (Nifurtimox); DB00945 (Aspirin); DB1083 (Orlistat) and DB11989 (Benznidazole). The first column 'PPP pathway proteins' lists the proteins associated with PPP pathways. The second column 'PMID' provides the PMID from PubMed for the paper from where evidence of protein association with the PPP was collected. The third column 'DB11820 (Nifurtimox)'	Supplementary Table 20.xlsx

		provides the binding energy of the Nifurtimox drug against the different proteins. The fourth column 'DB00945 (Aspirin)' provides the binding energy of Aspirin against the different proteins. The fifth column 'DB01083 (Orlistat)' provides the binding energy of Orlistat against the different proteins. The last column 'DB11989 (Benznidazole)' provides the binding energy of Benznidazole drug against the different proteins.	
21	Supplementary Table 21	**Supplementary Table 21: The binding affinity of benznidazole with 19,523 human proteins.** This table has 8 columns: Entry, Entry name, Status, Protein names, Gene names, Organism, Length, and DB11989. The first column 'Entry' list the protein IDs; the second column 'Entry name' list the name provided for each entry in the Alpha-fold database; the third column 'Status' provides the information on review status of each protein entry; the fourth column 'Protein names' list the names of each protein; the fifth column 'Gene names' list the name of the genes that encode the protein; the sixth column 'Organism' list the source organism of the protein; seventh column 'Length' list the length of each protein. The last column 'DB11989' lists benznidazole's binding affinity against each protein.	Supplementary Table 21.xlsx
22	Supplementary Table 22	**Supplementary Table 22: The binding affinity of 1516 FDA-approved drugs with the crystal structure of PGF (PDB: 2F38).** This table has 2 columns: Ligand and Binding energy. The first column 'Ligand' list the drug IDs and second column 'Binding Affinity' list the binding energy of each drug against prostaglandin F synthase (2F38).	Supplementary Table 22.xlsx
23	Supplementary Table 23	**Supplementary Table 23 a: The binding affinity of *T.cruzi* solved protein structures (targets) with nifurtimox, aspirin, orlistat and benznidazole.** This table has information in 5 columns: Protein ID, DB11820 (Nifurtimox), DB00945 (Aspirin), DB01083 (Orlistat) and DB11989 (Benznidazole). The first column 'Protein ID' provides the NCBI accession ID of the protein targets; The second column 'DB11820 (Nifurtimox)' provides the binding affinity (kcal/mol) of the protein target docked against Nifurtimox; The second column 'DB00945 (Aspirin)' provides the binding affinity (kcal/mol) of the protein target docked against Aspirin; The fourth column 'DB01083 (Orlistat)' provides the binding affinity (kcal/mol) of the protein target docked against Orlistat; The fifth column 'DB11989 (Benznidazole)' provides the binding affinity (kcal/mol) of the protein target docked against Benznidazole.	Supplementary Table 23.xlsx

		**Supplementray Table 23 b1: The molecular docking statistics of each drug docked against 5,004 *T. cruzi* CL Brener solved protein structures.** This table has information in 2 columns for each drug: Metric and Values. The first column 'Metric' provides the name of the metric measured to know the overall results of the drug when docked against all the protein structures; The second column provides the measured value of each metric in kcal/mol.	
		**Supplementray Table 23 b2: List of protein target with highest binding affinity for each drug.** This table lists the protein target with the highest binding affinity out of the total 5,004 *T. cruzi* CL Brener solved protein structures for each drug. The protein name, binding affinity, residure, NCBI protein accession ID, and NCBI gene accession ID is provided for each protein target.	
		**Supplementary Table 23 c: The top five ranking *T. cruzi* protein targets for nifurtimox, aspirin, orlistat and benznidazole.** This table has information in 3 columns for each drug: Protein ID, Score, and Protein name. The first column 'Protein ID' provides the NCBI accession ID of the protein targets; The second column 'Score' provides the binding affinity (kcal/mol) of the protein target when docked against the respective drug; The third column 'Protein name' provides the name of the top 5 protein targets for each drug.	
		**Supplementary Table 23 d: Results of Welch's T-Test applied to the binding affinity for different pairs of drug molecules.** This table has information in 5 columns: Student T-Test, T-value, 95 percent confidence interval, Sample Estimates and Mean of the Differences. The first column 'Student T-Test' indicates the names of the drugs (drug pair) for which the binding affinities are tested; The second column 'T-value' provides the T-value on the basis of the binding affinities of the two drugs to the *T. cruzi* protein structures; The third column '95 percent confidence interval' provides the 95 percent confidence interval on the basis of the binding affinities of the two drugs to the *T. cruzi* protein structures; The third column 'Sample Estimates' provides the sample estimates on the basis of the binding affinities of the two drugs to the *T. cruzi* protein structures	
24	Supplementary Table 24	**Supplementary Table 24 a: The binding affinity of *Plasmodium falciparum* with Benznidazole (DB11989).** This table has 3 columns. The first column 'Protein ID of *P. falciparum* proteins' provides the PDB ID of *P. falciparum* proteins; The second column 'Binding affinity (kcal/mol)' is the binding	Supplementary Table 24.xlsx

Commented [1]: remaining

		affinity of *P. falciparum* proteins when docked against Benznidazole (DB11989) drug; The third column 'Name of the protein' provides the name of the *P. falciparum* protein.	
		**Supplementary Table 24 b: The binding affinity of *Mycobacterium tuberculosis* with Benznidazole (DB11989).** This table has 3 columns. The first column 'Protein ID of *M. tuberculosis* proteins' provides the PDB ID of *M. tuberculosis* proteins; The second column 'Binding affinity (kcal/mol)' is the binding affinity of *M. tuberculosis* proteins when docked against Benznidazole (DB11989) drug; The third column 'Name of the protein' provides the name of the *M. tuberculosis* protein.	
		**Supplementary Table 24 c: The binding affinity of *Leishmania donovani* with Benznidazole (DB11989).** This table has 3 columns. The first column 'Protein ID of *Leishmania donovani* proteins' provides the PDB ID of *L. donovani* proteins; The second column 'Binding affinity (kcal/mol)' is the binding affinity of *L. donovani* proteins when docked against Benznidazole (DB11989) drug; The third column 'Name of the protein' provides the name of the *L. donovani* protein	
		**Supplementary Table 24 d: The binding affinity of *Salmonella typhi* with Benznidazole (DB11989).** This table has 3 columns. The first column 'Protein ID of *Salmonella typhi* proteins' provides the PDB ID of *S. typhi* proteins; The second column 'Binding affinity (kcal/mol)' is the binding affinity of *S. typhi* proteins when docked against Benznidazole (DB11989) drug; The third column 'Name of the protein' provides the name of the *S. typhi* protein	
		**Supplementary Table 24 e: Results of Welch's T-Test applied to the binding affinity of *T. cruzi* solved structures and other pathogens.** This table has information in 4 columns: Student T-Test, T-value, 95 percent confidence interval, and Sample Estimates. The first column 'Student T-Test' indicates the name of the pathogen for which the binding affinities are tested against *T. cruzi* solved structures; The second column 'T-value' provides the T-value on the basis of the binding affinities of *T. cruzi* solved structures and the pathogen; The third column '95 percent confidence interval' provides the 95 percent confidence interval on the basis of the binding affinities of *T. cruzi* solved structures and the pathogen; The fourth column 'Sample Estimates' provides the sample estimates on the basis of the binding affinities of *T. cruzi* solved structures and the pathogen	

25	Supplementary Table 25	**Supplementary Table 25: The binding affinity of FDA-approved drug molecules docked against Tc24.** This table has information in 2 columns: Ligand, and Affinity (kcal/mol). The first column 'Ligand' provides the DrugBank ID of the drug molecules docked against Tc24; The second column 'Affinity (kcal/mol)' provides the strength of the interaction between the drug molecule and Tc24 that bind reversibly.	Supplementary Table 25.xlsx
26	Supplementary Table 26	**Supplementary Table 26: Docking results of Benznidazole and Nifurtimox against Tc24 protein using AutoDock Vina.** This table has information in 5 columns: Drug name, Mode (Top 30), Affinity (kcal/mol), Distance from the best mode (rmsd l.b.), and Distance from the best mode (rmsd u.b.). The first column 'Drug name' provides the name of the drug docked against Tc24; The second column Mode (Top 3) provides the orientation of the ligand relative to the receptor as well as the conformation of the ligand and receptor when bound to each other; The third column Affinity (kcal/mol) provides the strength of the interaction between the drug molecule and Tc24 that bind reversibly; The fourth and fifth columns 'Distance from the best mode (rmsd l.b.)' and 'Distance from best mode (rmsd u.b.)' provides the distance of that particular mode from the best mode in terms of Root Mean Square Deviation (RMSD) - upper bound and lower bound.	Supplementary Table 26 .xlsx
27	Supplementary Table 27	**Supplementary Table 27 a: Simple parameters of *T.cruzi* pathways map determined using the network analyzer Cytoscape plugin.** This table has information in 2 columns: Type and Statistics. The first column 'Type' provides the type of the parameter; The second column 'Statistics' provides the value of the parameter measured using Cytoscape.	Supplementary Table 27.docx
		**Supplementary Table 27 b: Simple parameters of *T.cruzi* molecular map determined using the network analyzer Cytoscape plugin.** This table has information in 2 columns: Type and Statistics. The first column 'Type' provides the type of the parameter; The second column 'Statistics' provides the value of the parameter measured using Cytoscape.	
		**Supplementary Table 27 c: Simple parameters of *T.cruzi* drug map determined using the network analyzer Cytoscape plugin.** This table has information in 2 columns: Type and Statistics. The first column 'Type' provides the type of the parameter; The second column 'Statistics' provides the value of the parameter measured using Cytoscape.	
28	Supplementary Table 28	**Supplementary Table 28: List of *T. cruzi* genes**	Supplementary Table 28.xlsx

		**(class: Trypanosomatidae) found from the TritrypDB database during Gene Ontology Analysis.** This table has information in 7 columns: Gene ID, source_id, organism, Genomic Location (Gene), Description, Gene Type and Input ID. The first column 'Gene ID' provides the ID of the enriched gene; The second column 'source_id' provides the source ID of the enriched gene; The third column 'Organism' provides the name of the organism to which the enriched gene belongs; The fourth column 'Gene Location (Gene)' provides the location of the enriched gene; The fifth column 'Description' describes the enriched gene ; The sixth column 'Gene Type' provides the type of the enriched gene; The seventh column 'Input ID' provides the input ID of the enriched gene.	
29	Supplementary Table 29	**Supplementary Table 29: Total list of enriched genes that belong to *Trypanosoma cruzi* species (*Trypanosoma cruzi* CL Brener Esmeraldo-like, and *Trypanosoma cruzi* CL Brener Non-Esmeraldo-like).** This table has information in 4 columns: Gene ID, source_id, organism, Genomic Location (Gene), Description, Gene Type, and Input ID. The first column 'Gene ID' provides the ID of the enriched gene; The second column 'source_id' provides the source ID of the enriched gene; The third column 'Organism' provides the name of the organism to which the enriched gene belongs; The fourth column 'Gene Location (Gene)' provides the location of the enriched gene; The fifth column 'Description' describes the enriched gene; The sixth column 'Gene Type' provides the type of the enriched gene; The seventh column 'Input ID' provides the input ID of the enriched gene.	Supplementary Table 29.xlsx
30	Supplementary Table 30	**Supplementary Table 30 a: List of enriched genes in Biological processes related to *Trypanosoma cruzi* CL Brener Non-Esmeraldo-like.** This table has information in 11 columns: ID; Name; Bgd count; Result count; Result gene list; Pct of bgd; Fold enrichment; Odds ratio; P-value; Benjamini; and Bonferroni. The first column 'ID' provides the ID of the BP; The second column 'Name' provides the name of the BP; The third column 'Bgd count' provides the number of genes with this BP in the genome; The fourth column 'Result count' provides the number of genes with this BP in our analysis; The fifth 'Result gene list' provides the names of the genes with this BP in our analysis; The sixth column 'Pct of Bgd' provides percent of genes with this BP in our analysis divided by the percent of genes with this BP in the genome; The seventh column 'Fold enrichment' provides the percent that are present in your analysis of the genes in the genome with this BP; The eigth	Supplementary Table 30.xlsx

		column 'Odds ratio' provides -The odds of the BP appearing in the gene list are the same as that for the background list; The ninth column 'P-value' provides the probability of seeing at least x number of genes out of the total n genes in the list annotated to the BP, given the proportion of genes in the whole genome that are annotated to that BP; The tenth column 'Benjamini' provides the Benjamini-Hochburg false discovery rate which is a method for controlling false discovery rates for type 1 errors; The eleventh column 'Bonferroni' provides the Bonferroni adjusted P-values which is a method for correcting significance based on multiple comparisons.	
		**Supplementary Table 30 b: List of enriched genes in molecular function related to *Trypanosoma cruzi* CL Brener Non-Esmeraldo-like.** This table has information in 11 columns: ID; Name; Bgd count; Result count; Result gene list; Pct of bgd; Fold enrichment; Odds ratio; P-value; Benjamini; and Bonferroni. The first column 'ID' provides the ID of the MF; The second column 'Name' provides the name of the MF; The third column 'Bgd count' provides the number of genes with this MF in the genome; The fourth column 'Result count' provides the number of genes with this MF in our analysis; The fifth 'Result gene list' provides the names of the genes with this MF in our analysis; The sixth column 'Pct of Bgd' provides percent of genes with this MF in our analysis divided by the percent of genes with this MF in the genome; The seventh column 'Fold enrichment' provides the percent that are present in your analysis of the genes in the genome with this MF; The eigth column 'Odds ratio' provides -The odds of the MF appearing in the gene list are the same as that for the background list; The ninth column 'P-value' provides the probability of seeing at least x number of genes out of the total n genes in the list annotated to the MF, given the proportion of genes in the whole genome that are annotated to that MF; The tenth column 'Benjamini' provides the Benjamini-Hochburg false discovery rate which is a method for controlling false discovery rates for type 1 errors; The eleventh column 'Bonferroni' provides the Bonferroni adjusted P-values which is a method for correcting significance based on multiple comparisons.	
		**Supplementary Table 30 c: List of enriched genes in cellular component related to *Trypanosoma cruzi* CL Brener Non-Esmeraldo-like.** This table has information in 11 columns: ID; Name; Bgd count; Result count; Result gene list; Pct of bgd; Fold enrichment; Odds ratio; P-value; Benjamini; and Bonferroni. The first column 'ID' provides the ID of the	

		CC; The second column 'Name' provides the name of the CC; The third column 'Bgd count' provides the number of genes with this CC in the genome; The fourth column 'Result count' provides the number of genes with this CC in our analysis; The fifth 'Result gene list' provides the names of the genes with this CC in our analysis; The sixth column 'Pct of Bgd' provides percent of genes with this CC in our analysis divided by the percent of genes with this CC in the genome; The seventh column 'Fold enrichment' provides the percent that are present in your analysis of the genes in the genome with this CC; The eigth column 'Odds ratio' provides -The odds of the CC appearing in the gene list are the same as that for the background list; The ninth column 'P-value' provides the probability of seeing at least x number of genes out of the total n genes in the list annotated to the CC, given the proportion of genes in the whole genome that are annotated to that CC; The tenth column 'Benjamini' provides the Benjamini-Hochburg false discovery rate which is a method for controlling false discovery rates for type 1 errors; The eleventh column 'Bonferroni' provides the Bonferroni adjusted P-values which is a method for correcting significance based on multiple comparisons.	
		**Supplementary Table 30 d: List of enriched genes in metabolic pathways related to *Trypanosoma cruzi* CL Brener Non-Esmeraldo-like.** This table has information in 11 columns: ID; Name; Bgd count; Result count; Result gene list; Pct of bgd; Fold enrichment; Odds ratio; P-value; Benjamini; and Bonferroni. The first column 'ID' provides the ID of the MP; The second column 'Name' provides the name of the MP; The third column 'Bgd count' provides the number of genes with this MP in the genome; The fourth column 'Result count' provides the number of genes with this MP in our analysis; The fifth 'Result gene list' provides the names of the genes with this MP in our analysis; The sixth column 'Pct of Bgd' provides percent of genes with this MP in our analysis divided by the percent of genes with this MP in the genome; The seventh column 'Fold enrichment' provides the percent that are present in your analysis of the genes in the genome with this MP; The eigth column 'Odds ratio' provides -The odds of the MP appearing in the gene list are the same as that for the background list; The ninth column 'P-value' provides the probability of seeing at least x number of genes out of the total n genes in the list annotated to the MP, given the proportion of genes in the whole genome that are annotated to that MP; The tenth column 'Benjamini' provides the Benjamini-Hochburg false discovery rate which is a method for controlling false discovery rates for type 1 errors; The eleventh column	

		'Bonferroni' provides the Bonferroni adjusted P-values which is a method for correcting significance based on multiple comparisons.	
31	Supplementary Table 31	**Suppelementary Table 31 a: List of enriched genes in biological process (BP) related to *Trypanosoma cruzi* CL Brener Esmeraldo-like.** This table has information in 11 columns: ID; Name; Bgd count; Result count; Result gene list; Pct of bgd; Fold enrichment; Odds ratio; P-value; Benjamini; and Bonferroni. The first column 'ID' provides the ID of the BP; The second column 'Name' provides the name of the BP; The third column 'Bgd count' provides the number of genes with this BP in the genome; The fourth column 'Result count' provides the number of genes with this BP in our analysis; The fifth 'Result gene list' provides the names of the genes with this BP in our analysis; The sixth column 'Pct of Bgd' provides percent of genes with this BP in our analysis divided by the percent of genes with this BP in the genome; The seventh column 'Fold enrichment' provides the percent that are present in your analysis of the genes in the genome with this BP; The eigth column 'Odds ratio' provides -The odds of the BP appearing in the gene list are the same as that for the background list; The ninth column 'P-value' provides the probability of seeing at least x number of genes out of the total n genes in the list annotated to the BP, given the proportion of genes in the whole genome that are annotated to that BP; The tenth column 'Benjamini' provides the Benjamini-Hochburg false discovery rate which is a method for controlling false discovery rates for type 1 errors; The eleventh column 'Bonferroni' provides the Bonferroni adjusted P-values which is a method for correcting significance based on multiple comparisons	Supplementary Table 31.xlsx
		**Supplementary Table 31 b: List of enriched genes in molecular function (MF) related to *Trypanosoma cruzi* CL Brener Esmeraldo-like.** This table has information in 11 columns: ID; Name; Bgd count; Result count; Result gene list; Pct of bgd; Fold enrichment; Odds ratio; P-value; Benjamini; and Bonferroni. The first column 'ID' provides the ID of the MF; The second column 'Name' provides the name of the MF; The third column 'Bgd count' provides the number of genes with this MF in the genome; The fourth column 'Result count' provides the number of genes with this MF in our analysis; The fifth 'Result gene list' provides the names of the genes with this MF in our analysis; The sixth column 'Pct of Bgd' provides percent of genes with this MF in our analysis divided by the percent of genes with this MF in the genome; The seventh column 'Fold enrichment' provides the percent that are present in your analysis	

		of the genes in the genome with this MF; The eigth column 'Odds ratio' provides -The odds of the MF appearing in the gene list are the same as that for the background list; The ninth column 'P-value' provides the probability of seeing at least x number of genes out of the total n genes in the list annotated to the MF, given the proportion of genes in the whole genome that are annotated to that MF; The tenth column 'Benjamini' provides the Benjamini-Hochburg false discovery rate which is a method for controlling false discovery rates for type 1 errors; The eleventh column 'Bonferroni' provides the Bonferroni adjusted P-values which is a method for correcting significance based on multiple comparisons.	
		**Supplementary Table 31 c:** List of enriched genes in cellular component related to *Trypanosoma cruzi* CL Brener Esmeraldo-like. This table has information in 11 columns: ID; Name; Bgd count; Result count; Result gene list; Pct of bgd; Fold enrichment; Odds ratio; P-value; Benjamini; and Bonferroni. The first column 'ID' provides the ID of the CC; The second column 'Name' provides the name of the CC; The third column 'Bgd count' provides the number of genes with this CC in the genome; The fourth column 'Result count' provides the number of genes with this CC in our analysis; The fifth 'Result gene list' provides the names of the genes with this CC in our analysis; The sixth column 'Pct of Bgd' provides percent of genes with this CC in our analysis divided by the percent of genes with this CC in the genome; The seventh column 'Fold enrichment' provides the percent that are present in your analysis of the genes in the genome with this CC; The eigth column 'Odds ratio' provides -The odds of the CC appearing in the gene list are the same as that for the background list; The ninth column 'P-value' provides the probability of seeing at least x number of genes out of the total n genes in the list annotated to the CC, given the proportion of genes in the whole genome that are annotated to that CC; The tenth column 'Benjamini' provides the Benjamini-Hochburg false discovery rate which is a method for controlling false discovery rates for type 1 errors; The eleventh column 'Bonferroni' provides the Bonferroni adjusted P-values which is a method for correcting significance based on multiple comparisons.	
		**Supplementary Table 31 d: List of enriched genes in molecular function related to *Trypanosoma cruzi* CL Brener Esmeraldo-like.** This table has information in 11 columns: ID; Name; Bgd count; Result count; Result gene list; Pct of bgd; Fold enrichment; Odds ratio; P-value; Benjamini; and Bonferroni. The first column 'ID' provides the ID of the MP; The second column 'Name' provides the name of	

		the MP; The third column 'Bgd count' provides the number of genes with this MP in the genome; The fourth column 'Result count' provides the number of genes with this MP in our analysis; The fifth 'Result gene list' provides the names of the genes with this MP in our analysis; The sixth column 'Pct of Bgd' provides percent of genes with this MP in our analysis divided by the percent of genes with this MP in the genome; The seventh column 'Fold enrichment' provides the percent that are present in your analysis of the genes in the genome with this MP; The eigth column 'Odds ratio' provides -The odds of the MP appearing in the gene list are the same as that for the background list; The ninth column 'P-value' provides the probability of seeing at least x number of genes out of the total n genes in the list annotated to the MP, given the proportion of genes in the whole genome that are annotated to that MP; The tenth column 'Benjamini' provides the Benjamini-Hochburg false discovery rate which is a method for controlling false discovery rates for type 1 errors; The eleventh column 'Bonferroni' provides the Bonferroni adjusted P-values which is a method for correcting significance based on multiple comparisons.	
