# Supplementary figures and images for: A Data-Driven Approach to Construct a Molecular Map of Trypanosoma cruzi to Identify Drugs and Vaccine Targets
Source: Vaccines (Basel). 2023 Jan 26;11(2):267. doi: 10.3390/vaccines11020267 (PMC9963959; doi:10.3390/vaccines11020267)

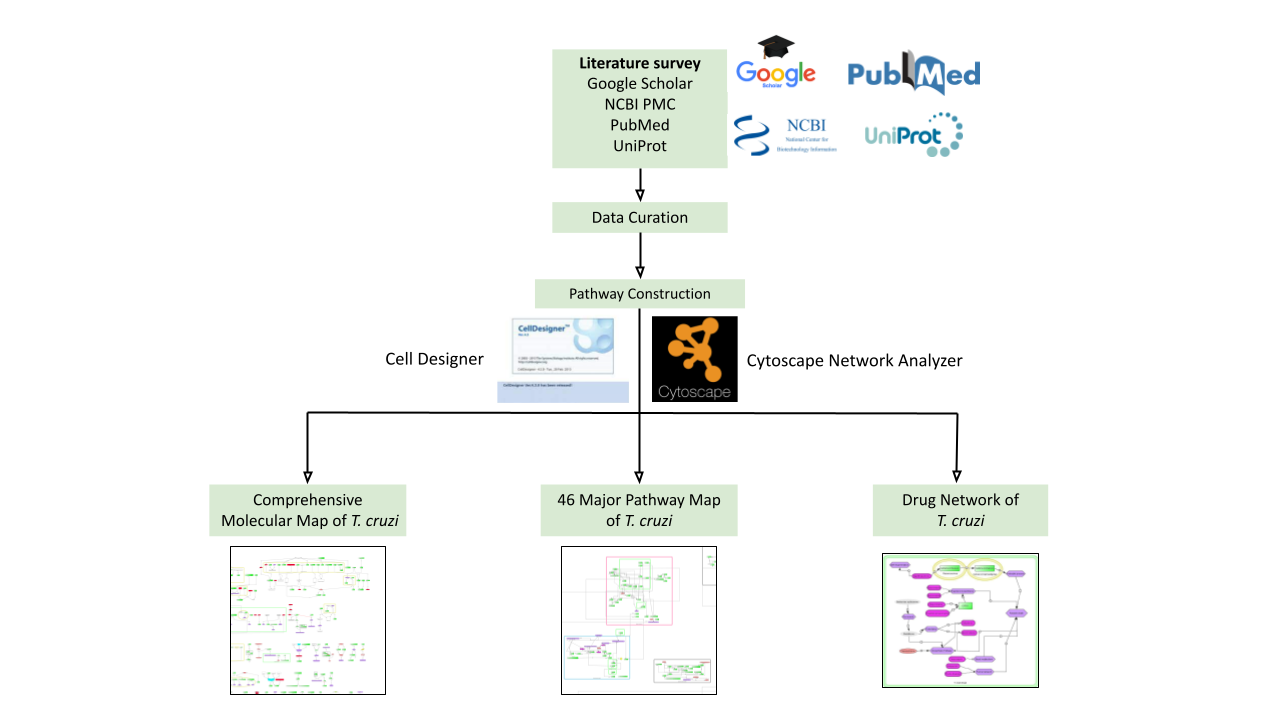

Supplement: Supplementary file 1 [file vaccines-11-00267-s001.zip › Supplementary Figures/Supplementary Figure 1.png]

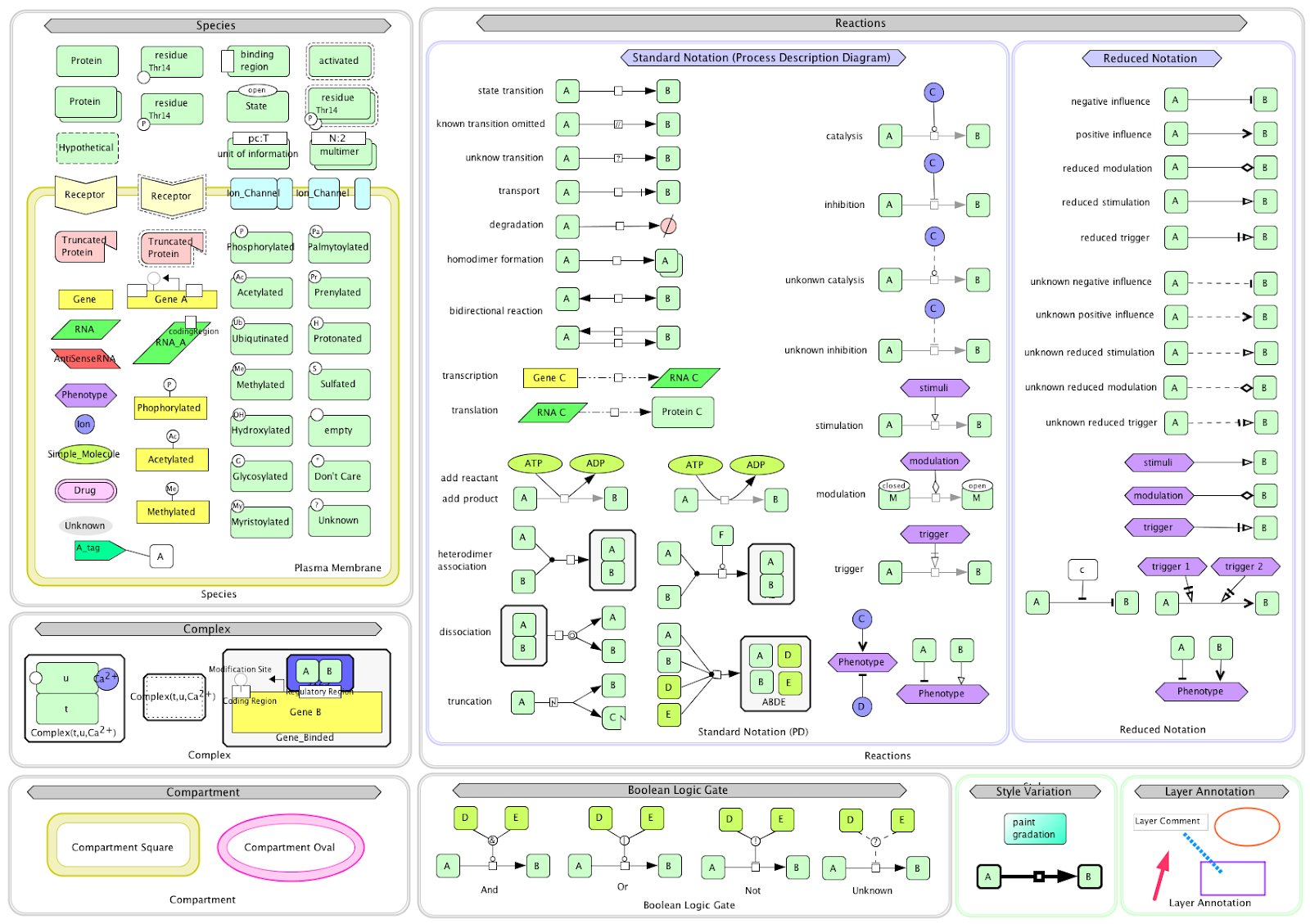

Supplement: Supplementary file 1 [file vaccines-11-00267-s001.zip › Supplementary Figures/Supplementary Figure 2.png]

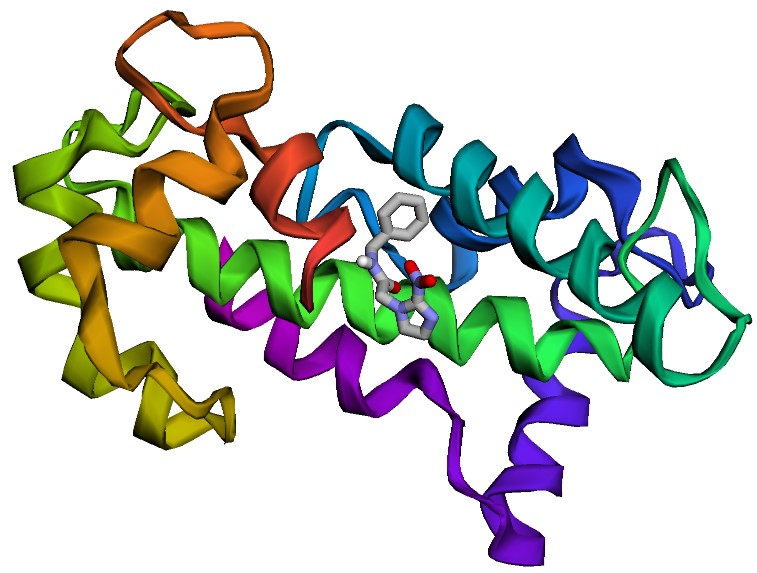

Supplement: Supplementary file 1 [file vaccines-11-00267-s001.zip › Supplementary Figures/Supplementary Figure 3/3(a).jpg]

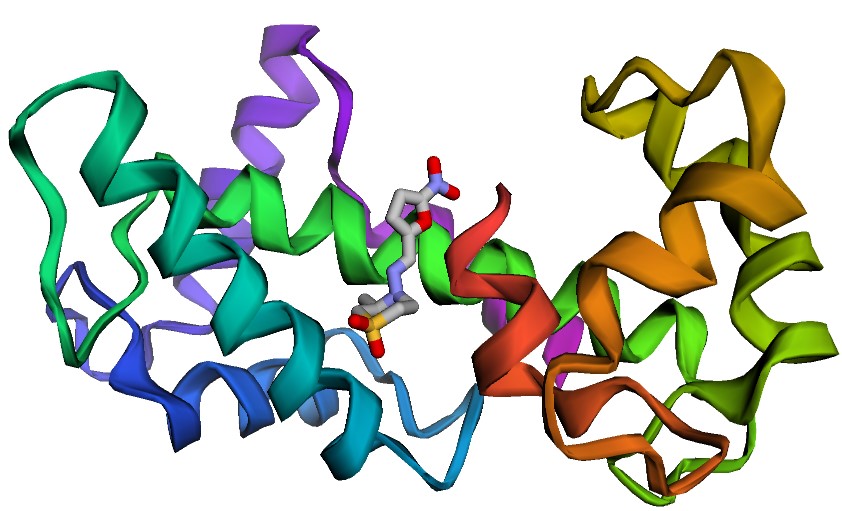

Supplement: Supplementary file 1 [file vaccines-11-00267-s001.zip › Supplementary Figures/Supplementary Figure 3/3(b).jpg]

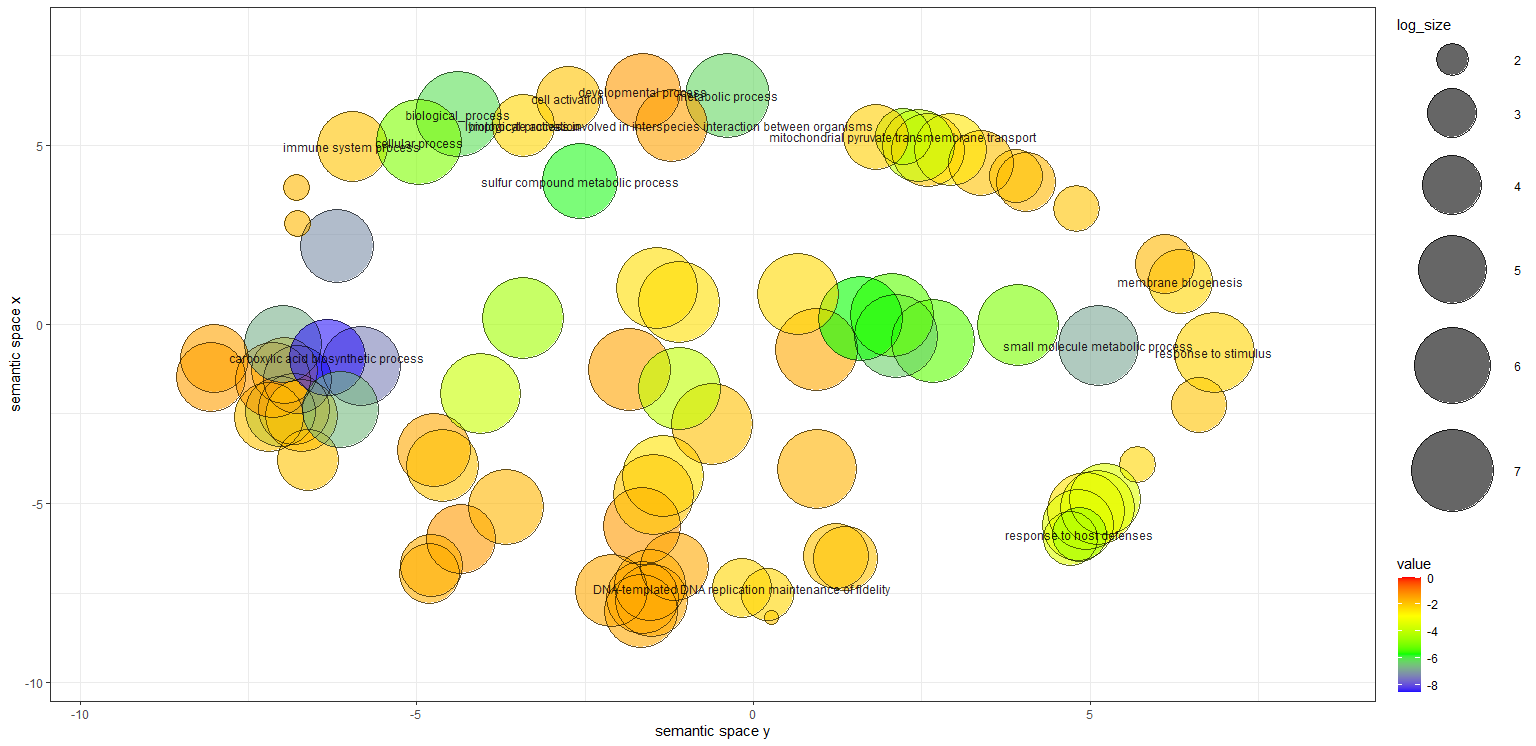

Supplement: Supplementary file 1 [file vaccines-11-00267-s001.zip › Supplementary Figures/Supplementary Figure 4/4A(a).tif]

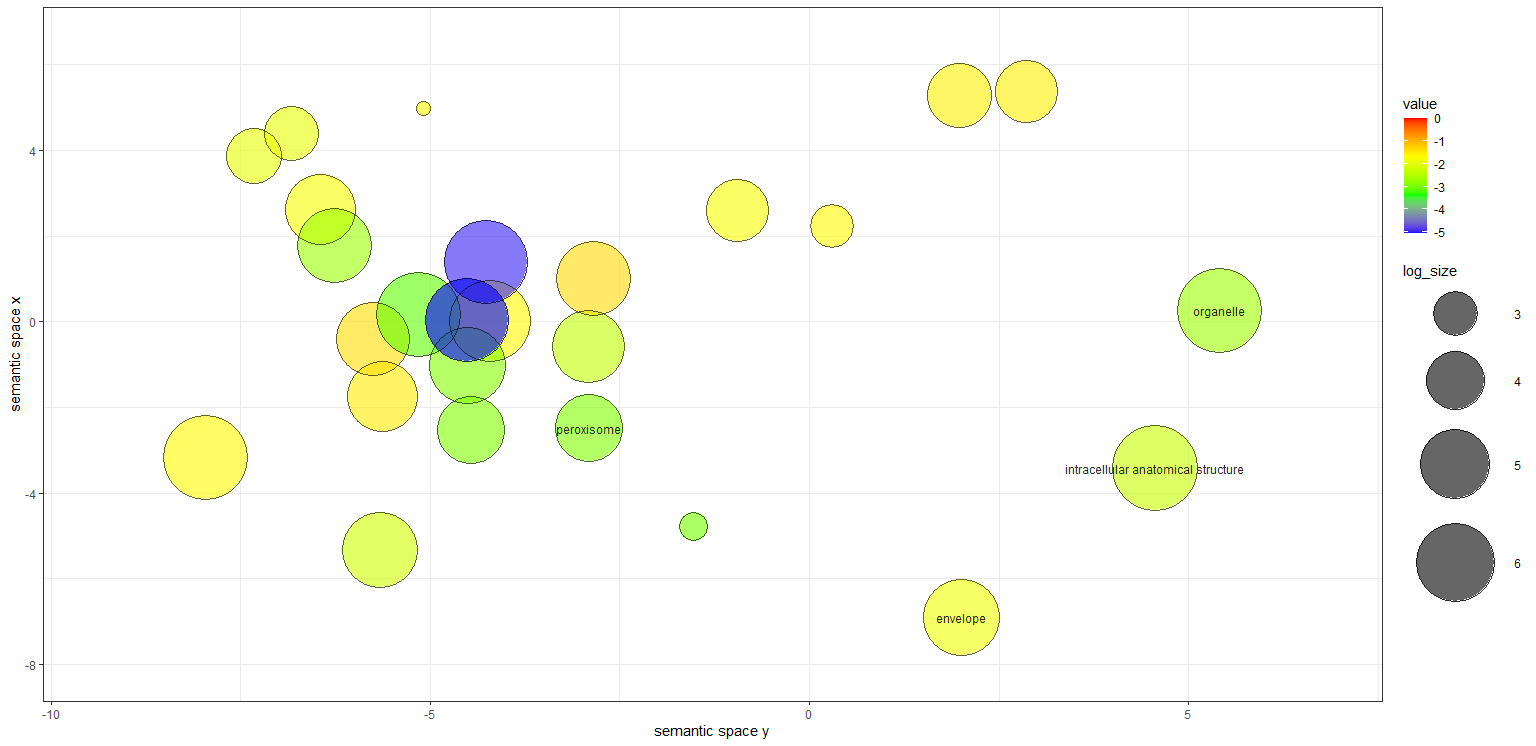

Supplement: Supplementary file 1 [file vaccines-11-00267-s001.zip › Supplementary Figures/Supplementary Figure 4/4A(b).tiff]

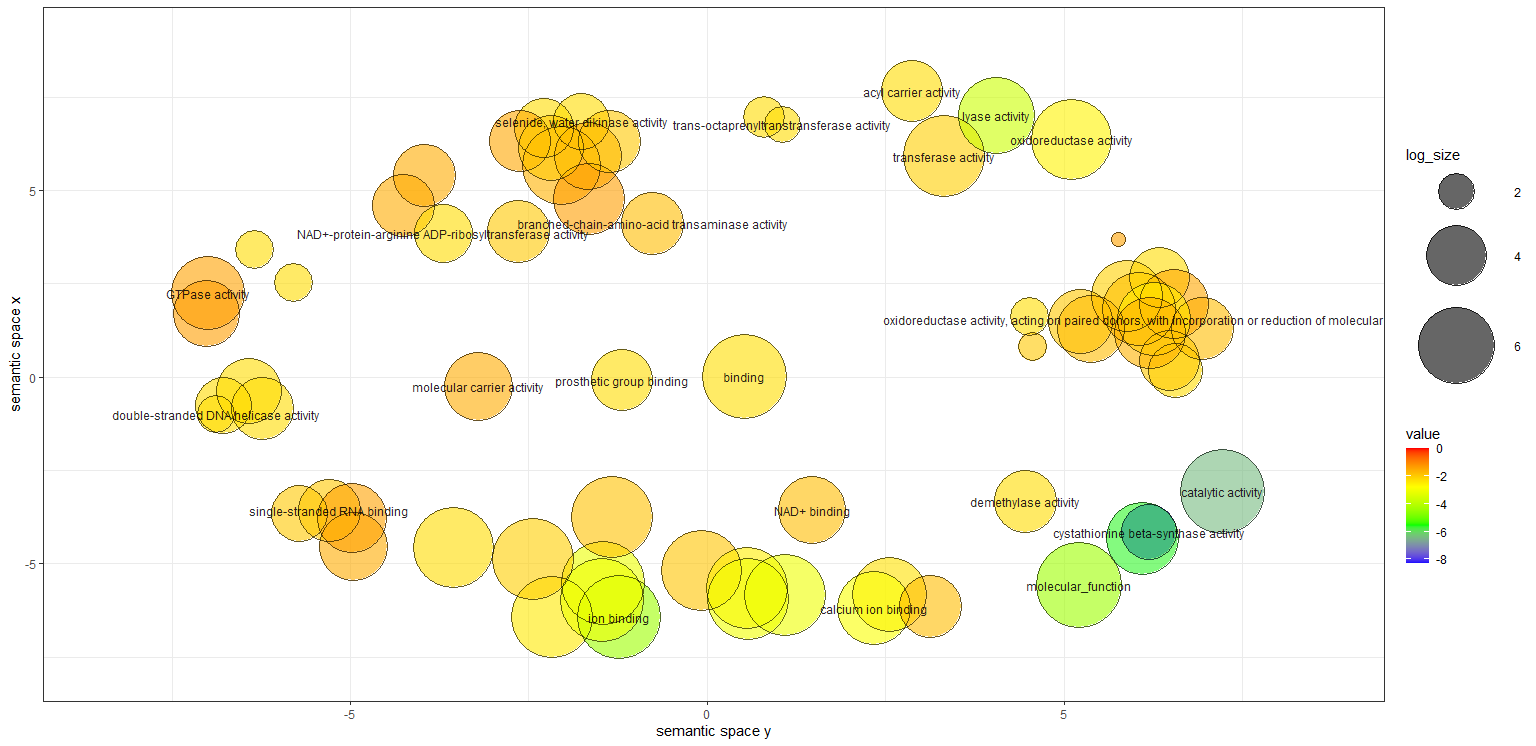

Supplement: Supplementary file 1 [file vaccines-11-00267-s001.zip › Supplementary Figures/Supplementary Figure 4/4A(c).tiff]

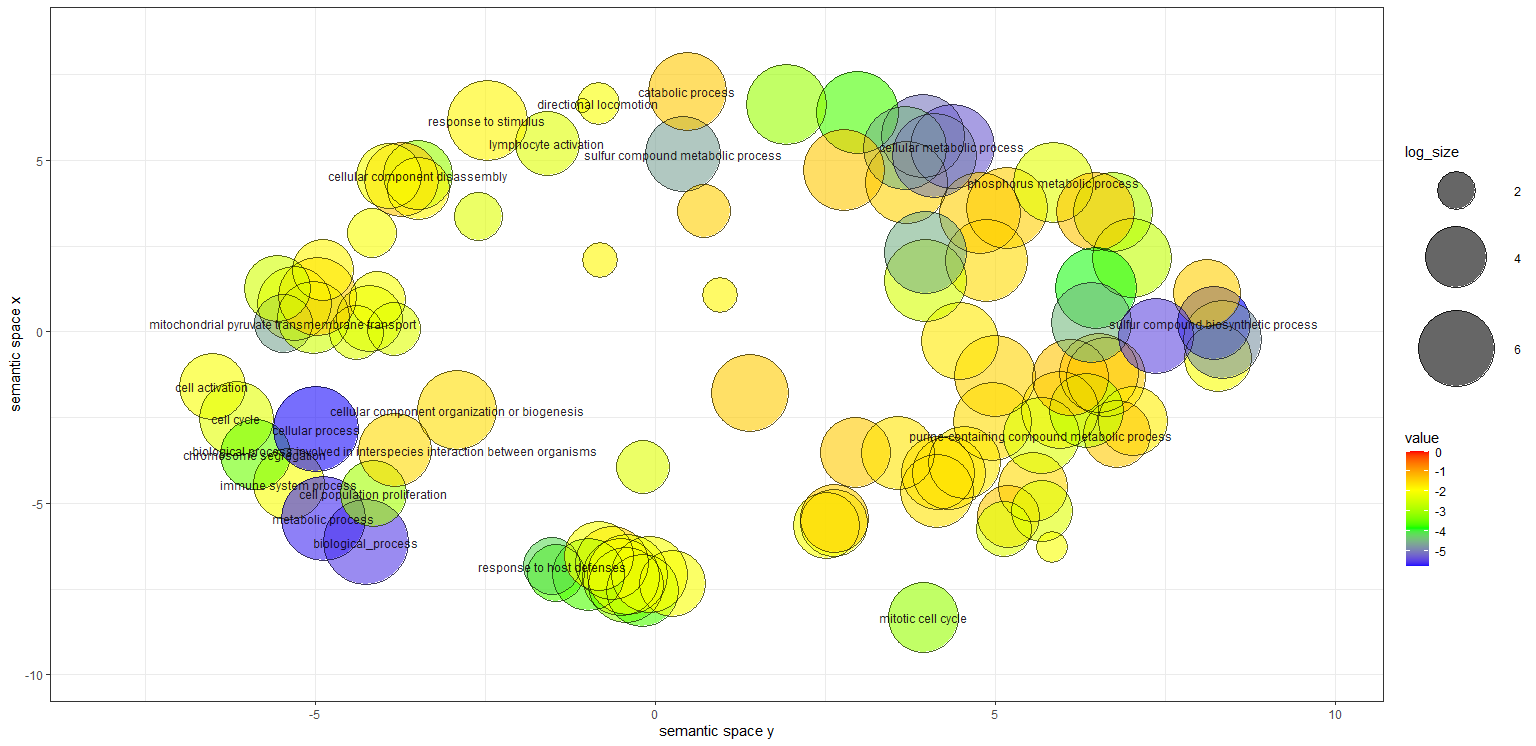

Supplement: Supplementary file 1 [file vaccines-11-00267-s001.zip › Supplementary Figures/Supplementary Figure 4/4B(a).tiff]

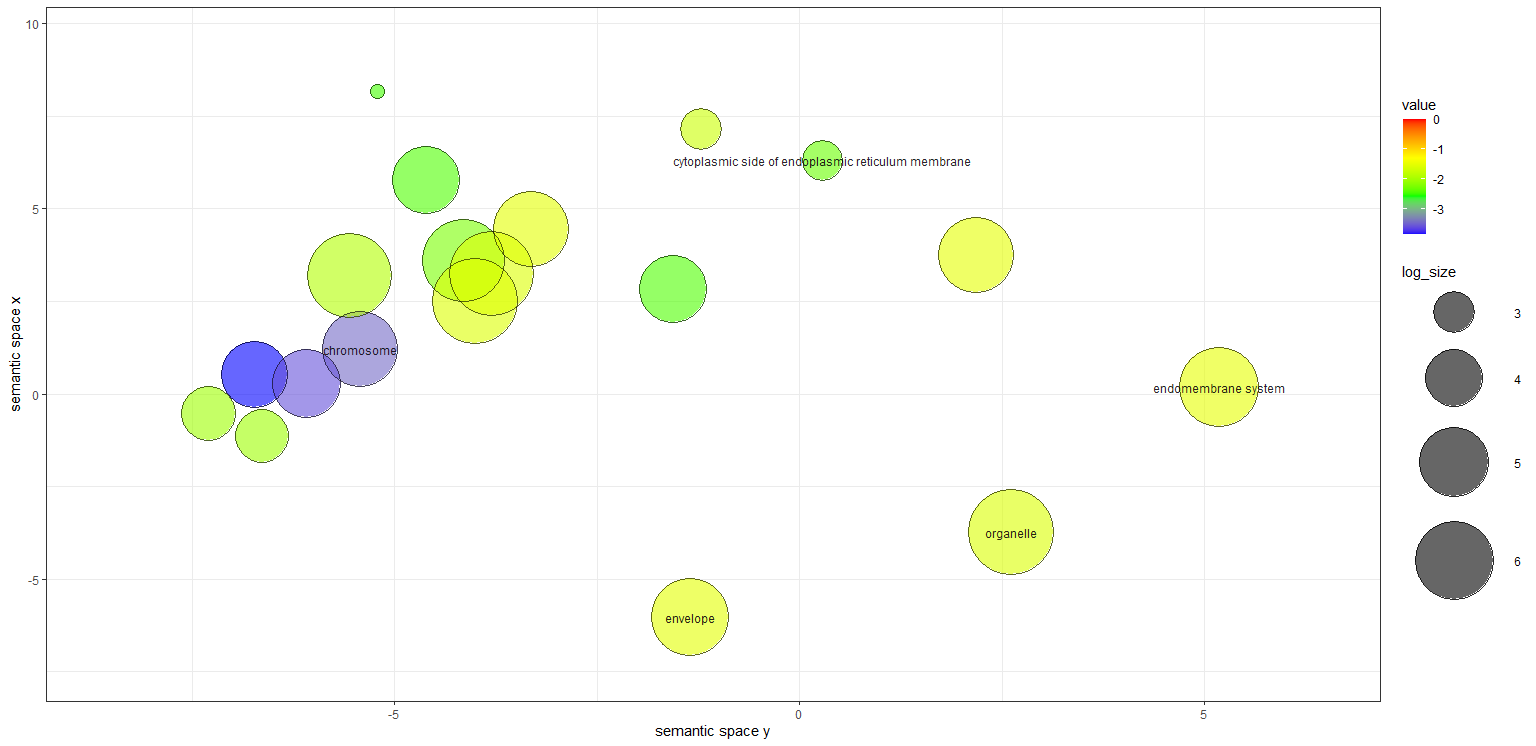

Supplement: Supplementary file 1 [file vaccines-11-00267-s001.zip › Supplementary Figures/Supplementary Figure 4/4B(b).tiff]

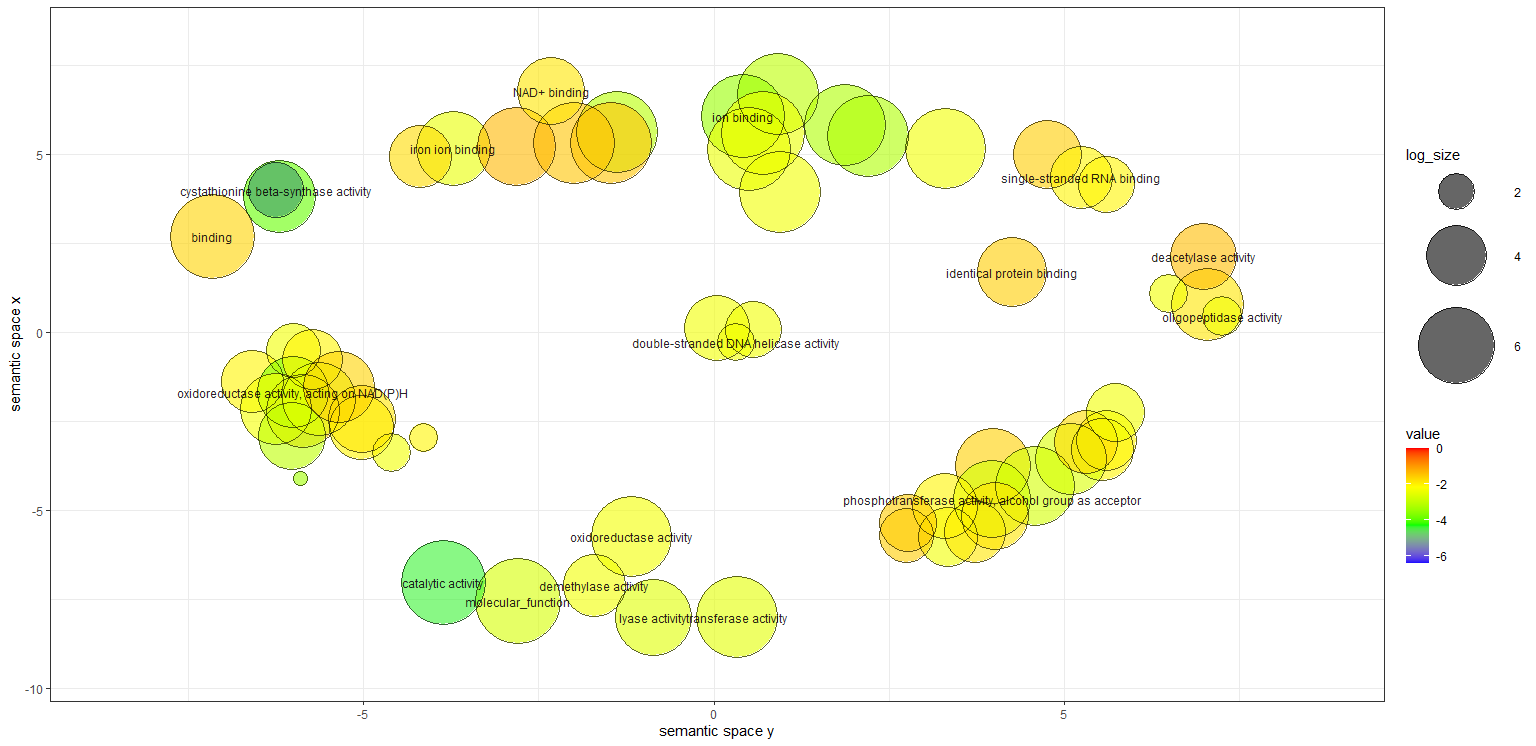

Supplement: Supplementary file 1 [file vaccines-11-00267-s001.zip › Supplementary Figures/Supplementary Figure 4/4B(c).tiff]

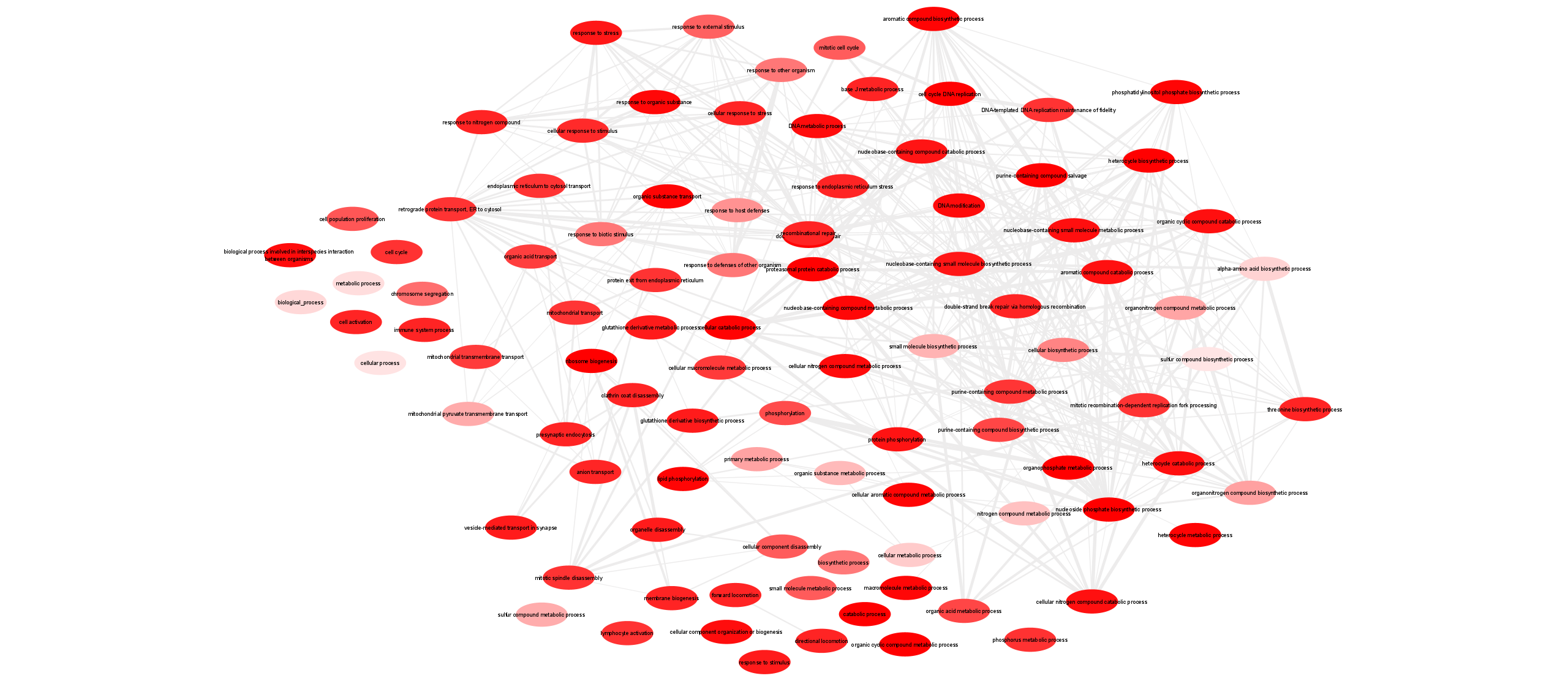

Supplement: Supplementary file 1 [file vaccines-11-00267-s001.zip › Supplementary Figures/Supplementary Figure 5/5A(a).png]

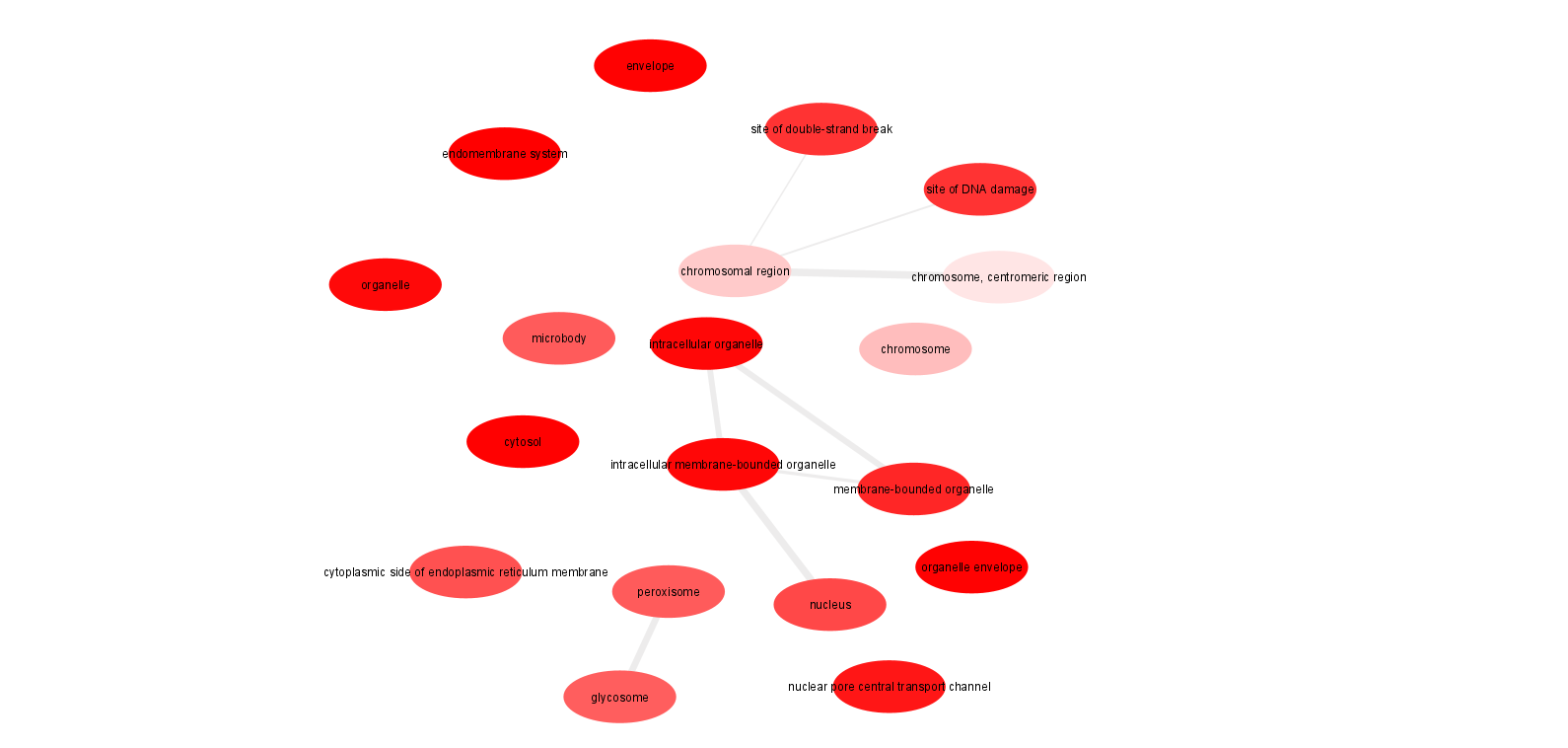

Supplement: Supplementary file 1 [file vaccines-11-00267-s001.zip › Supplementary Figures/Supplementary Figure 5/5A(b).png]

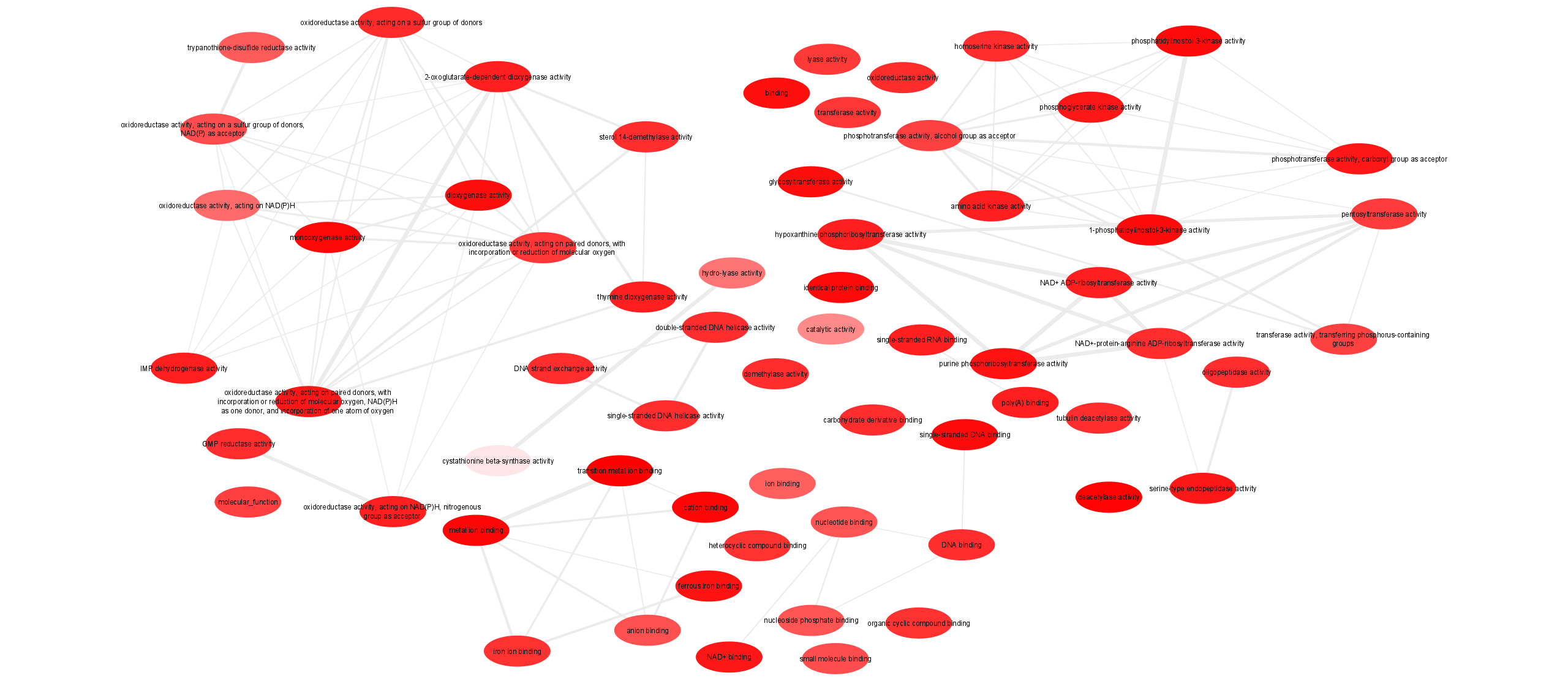

Supplement: Supplementary file 1 [file vaccines-11-00267-s001.zip › Supplementary Figures/Supplementary Figure 5/5A(c).png]

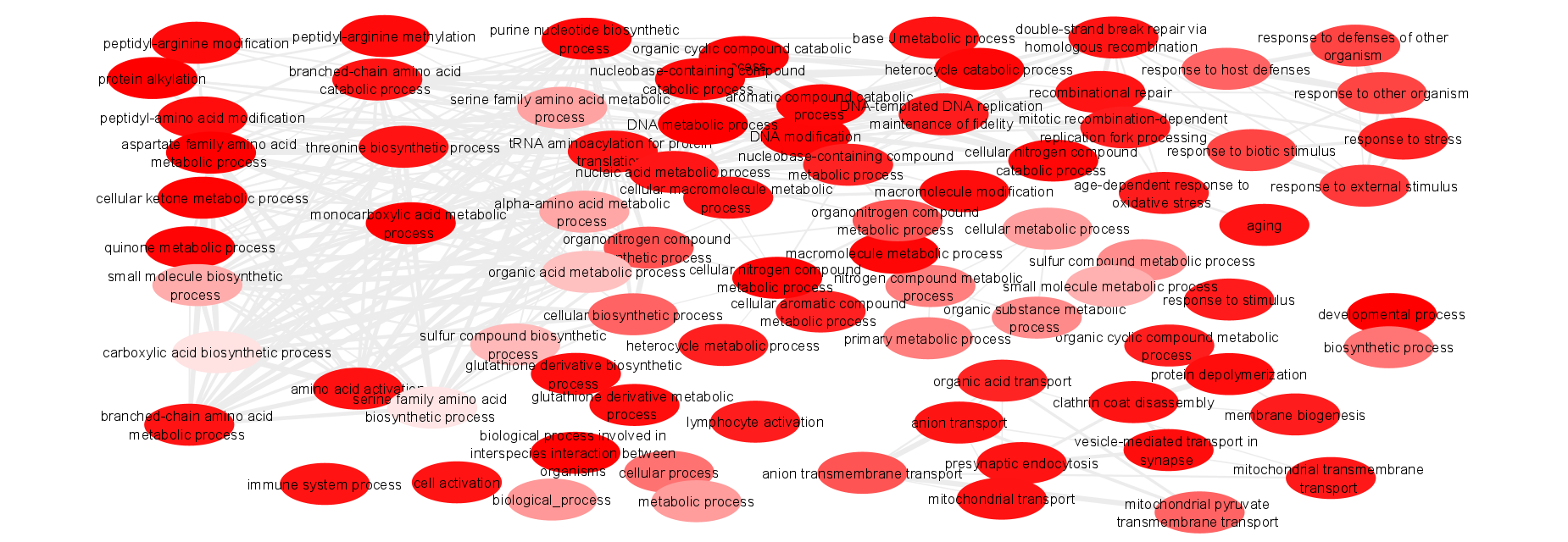

Supplement: Supplementary file 1 [file vaccines-11-00267-s001.zip › Supplementary Figures/Supplementary Figure 5/5B(a).png]

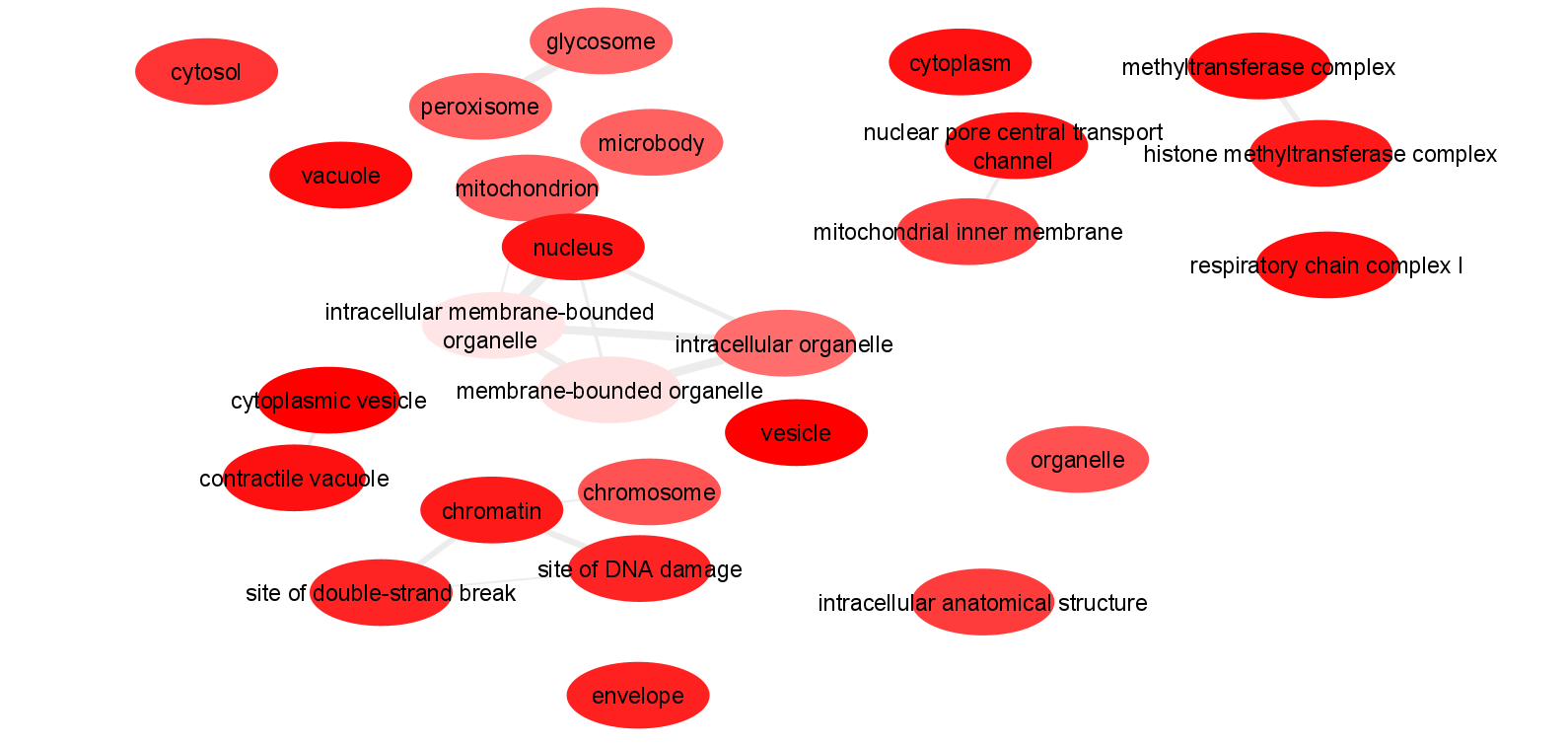

Supplement: Supplementary file 1 [file vaccines-11-00267-s001.zip › Supplementary Figures/Supplementary Figure 5/5B(b).png]

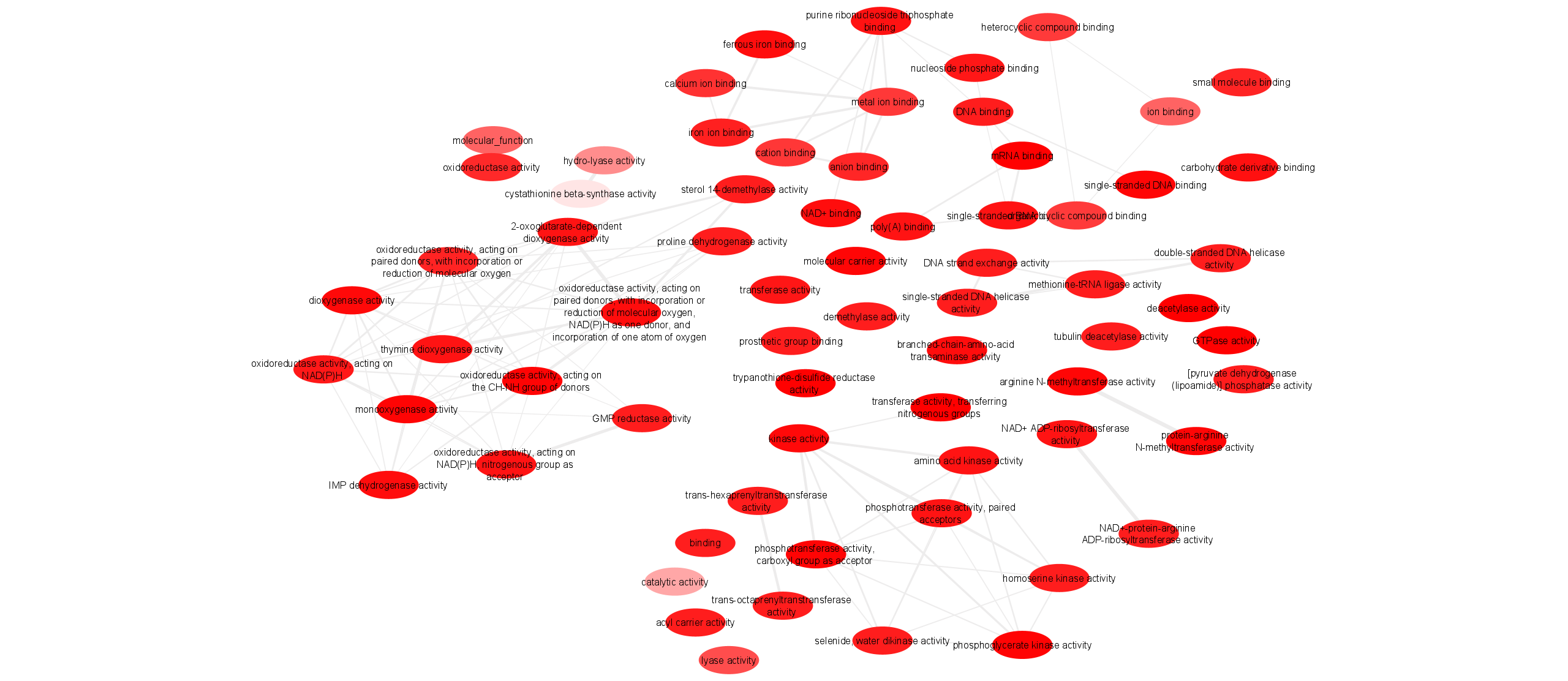

Supplement: Supplementary file 1 [file vaccines-11-00267-s001.zip › Supplementary Figures/Supplementary Figure 5/5B(c).png]

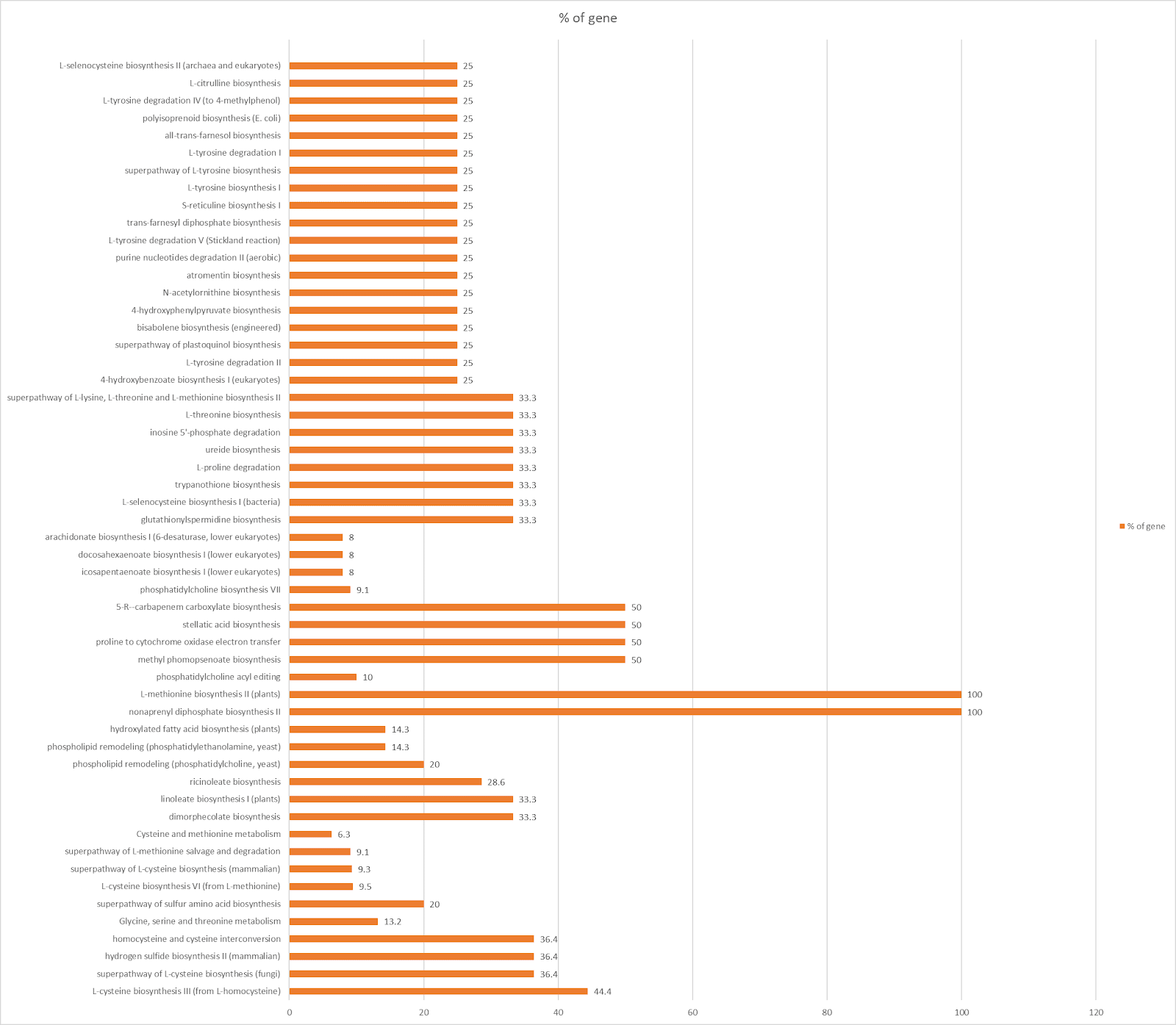

Supplement: Supplementary file 1 [file vaccines-11-00267-s001.zip › Supplementary Figures/Supplementary Figure 6/6(a).png]

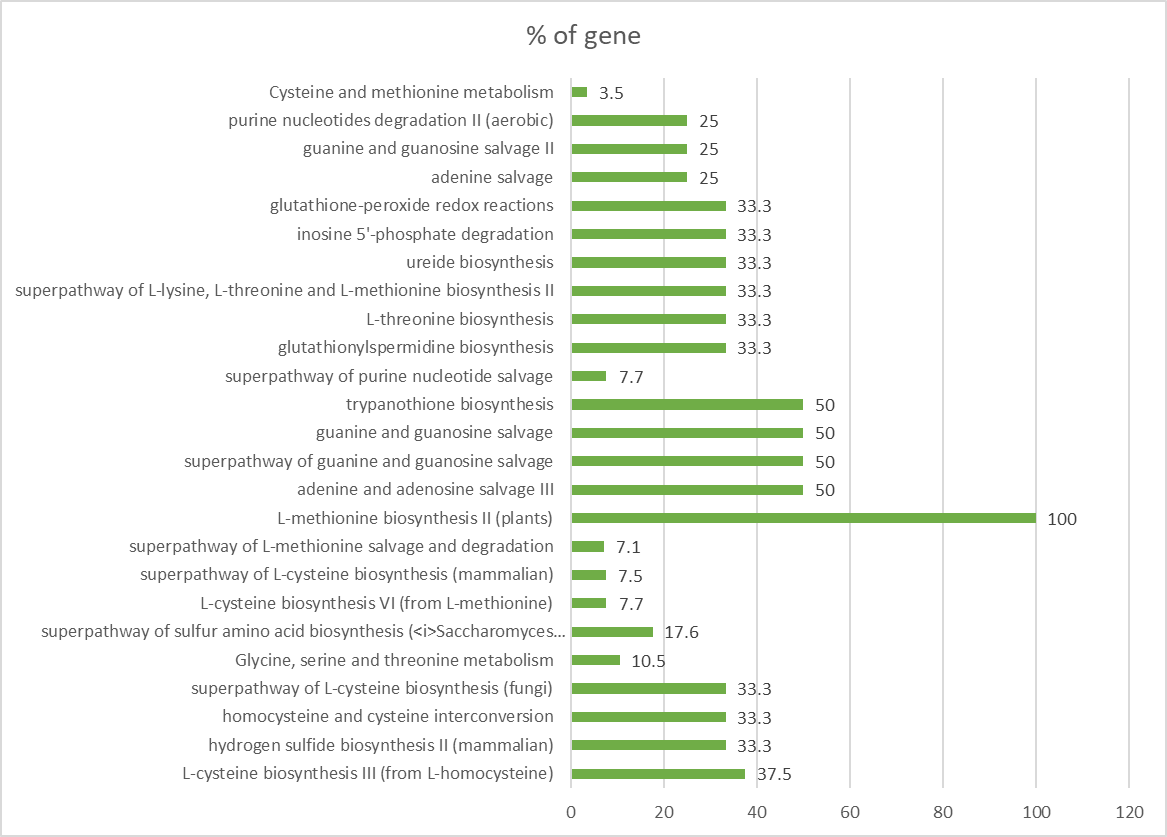

Supplement: Supplementary file 1 [file vaccines-11-00267-s001.zip › Supplementary Figures/Supplementary Figure 6/6(b).png]

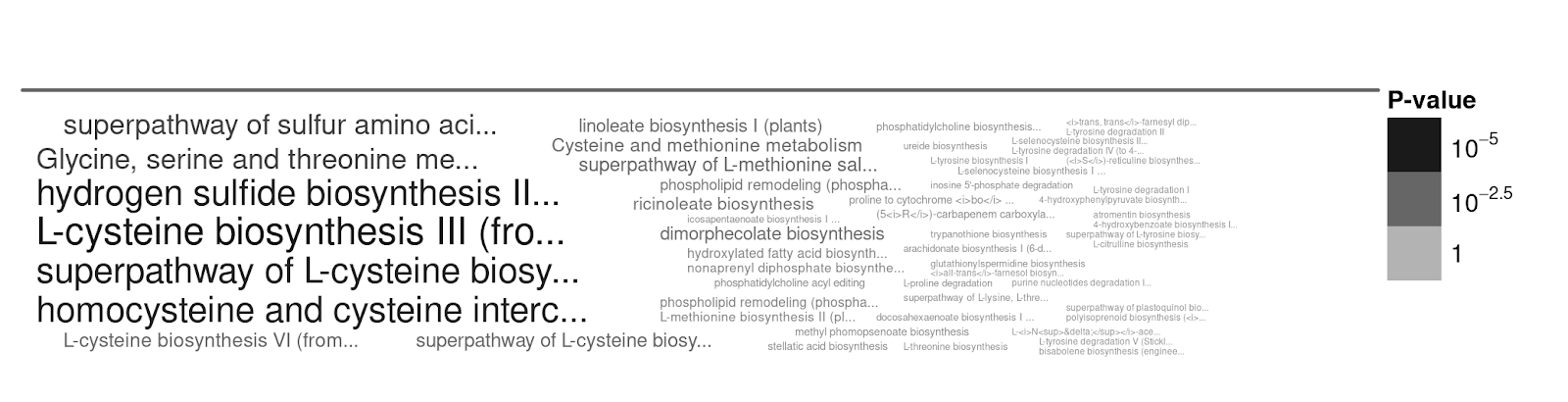

Supplement: Supplementary file 1 [file vaccines-11-00267-s001.zip › Supplementary Figures/Supplementary Figure 6/6(c)(1).png]

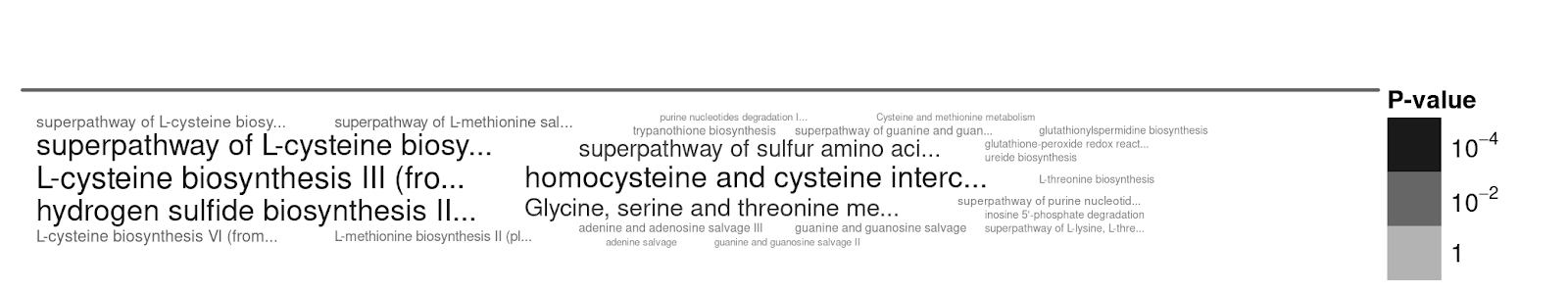

Supplement: Supplementary file 1 [file vaccines-11-00267-s001.zip › Supplementary Figures/Supplementary Figure 6/6(c)(2).png]
